# Coding and Noncoding Uterine Small Extracellular Vesicle Content Differs in the Early Stages of Pregnancies Produced by Artificial Insemination and In Vitro Fertilization in Cattle

**DOI:** 10.1002/mrd.70132

**Published:** 2026-07-06

**Authors:** Amanda de Oliveira Furlan, Alessandra Bridi, Jéssica Nora Drum, Liza Margareth Medeiros de Carvalho Sousa, Mariana Cordeiro Almeida, Juliana de Souza Felix, Luana Araujo Zutin, Isabelle Alexandrino dos Santos, Flávia Regina Florencio de Athayde, Natália Francisco Scaramele, Camila Azzolin de Souza, Paula de Carvalho Papa, Juliano Coelho da Silveira, Roberto Sartori Filho, Danila Barreiro Campos, Flavia Lombardi Lopes

**Affiliations:** ^1^ Department of Production and Animal Health, School of Veterinary Medicine São Paulo State University (UNESP) Araçatuba São Paulo Brazil; ^2^ Department of Veterinary Medicine, Faculty of Animal Sciences and Food Engineering University of São Paulo (USP) Pirassununga São Paulo Brazil; ^3^ Department of Animal Science, “Luiz de Queiroz” College of Agriculture (ESALQ) University of São Paulo Piracicaba São Paulo Brazil; ^4^ Polytechnic Institute UniLaSalle School of Veterinary Medicine Rouen France; ^5^ Department of Veterinary Sciences, Agricultural Science Center Federal University of Paraíba (UFPB) Areia Brazil

**Keywords:** bovine, IVF‐ET, maternal‐fetal crosstalk, ncRNA, small extracellular vesicle

## Abstract

Establishing a successful pregnancy relies on effective communication between the mother and fetus. Small extracellular vesicles (sEVs) are pivotal in facilitating intercellular communication, transferring messenger RNA (mRNA), noncoding RNA (ncRNA), and proteins. Considering the prevalence of in vitro fertilization (IVF) in bovine production, despite its lower birth rate, we aimed to evaluate whether there are differences in transcript content in sEVs between pregnancies from artificial insemination fixed‐time (FTAI) and in vitro fertilization—embryo transfer (IVF‐ET). Uterine fluid was collected on Days 18 and 32 from AI‐ and IVF‐derived pregnancies in beef cattle. sEVs were isolated, and total RNA was extracted for transcriptome and microtranscriptome analysis. Differences in transcript abundance were greatest when comparing Days 18 and 32 within the IVF‐ET, suggesting that temporal changes in transcripts are highly influenced by reproductive technique. Gene set enrichment analysis showed 22 gene sets in IVF‐ET18 x FTAI18, with mRNAs involved in embryonic pre‐adhesion that may impair embryo fixation. In IVF‐ET32 x IVF‐ET18, target genes of differentially expressed (DE) microRNAs (miRNAs) were enriched in endocytosis pathway, while target genes of DE lncRNAs were enriched in complement and coagulation cascades pathway. In summary, sEVs derived from FTAI and IVF‐ET pregnancies have different coding and noncoding transcript content, suggesting failures in maternal‐fetal communication post‐IVF.

## 1 Introduction

Assisted reproductive technologies, such as artificial insemination (AI) and in vitro fertilization (IVF), are widely used across a few species and have been increasingly adopted, with global bovine in vitro embryo production increasing by 15.8% in 2023 and a further 8.0% in 2024 according to IETS annual reports (Viana 2024). Its use in IVF‐ET has gained prominence in livestock production because it enables the production of a greater number of offspring in a shorter period, thereby accelerating genetic gain in herds (Lafontaine et al. 2023). Notwithstanding, IVF is associated with a higher pregnancy loss rate between Days 32 and 62 compared to AI, with losses of 15.1% and 4.7%, respectively (Crowe et al. 2024), which translates into considerable financial loss for producers, with estimated embryo costs ranging from $100–$200 per transfer and additional replacement heifer costs reaching up to $109 per cow annually in cases of failed pregnancies (Ferreira et al. 2021). Crosstalk at the maternal‐fetal interface involves the interaction between different cell types and plays a fundamental role in the establishment and maintenance of pregnancy (Lash 2015). Thus, failure in the establishment of proper fetal‐maternal crosstalk is believed to play a major role in the observed pregnancy rates (Reza et al. 2019).

Small extracellular vesicles (sEVs), generally 30–150 nm in diameter and defined as vesicles smaller than 200 nm, are released by most cells in response to physiological or pathological stimuli (Cavallaro et al. 2021). sEVs derive from multivesicular bodies (MVBs), which are late endosomes containing intraluminal vesicles (ILVs). sEVs carry proteins, lipids, metabolites, and nucleic acids that reflect not only their cellular origin but also the physiological state of the parent cell. Consequently, EVs released from the same cell type may exhibit distinct molecular cargo under different physiological conditions. sEVs are specific to their cellular origin, and they can carry and deliver their loads to cells in their vicinity or away from their origin (Li et al. 2019). They are essential to pregnancy, participating in the processes of implantation, migration, and invasion of trophoblasts (Nowak 2016); modifications in their composition and concentration have been associated to gestational diseases (Zhang et al. 2020). sEVs also participate in maternal‐fetal communication, starting at the preimplantation stage (Salamonsen et al. 2009). Prior to and throughout pregnancy, uterine endometrial cells and trophoblasts secrete sEVs that play a crucial role in facilitating this communication (Hedlund et al. 2009; Taylor et al. 2006).

Noncoding RNAs (ncRNAs) are not translated into protein and can be transported via sEVs (Joshi and Rajender 2020). MicroRNAs (miRNAs), a class of small ncRNAs, contain about 19–25 nucleotides and function through posttranscriptional regulation of mRNAs, and their expression is modulated in response to a multitude of stimuli (Ambros 2004; Costa et al. 2012). Due to their expression in reproductive tissues and data from transgenic mice and cell models in other species (Lei et al. 2010), miRNAs have been shown to regulate folliculogenesis (Liang et al. 2017) to uterine functions during peri‐ and postimplantation periods (Xu et al. 2013). Using epithelial cells from the oviduct of inseminated cows, researchers have observed that maternal‐fetal communication begins in the oviduct and that the presence of the embryo modulates the content of miRNAs present in sEVs (Mazzarella et al. 2021). Following IVF in cattle, it was also found that the miRNA content of embryo‐derived sEVs isolated from embryo culture media varied according to developmental stage, when comparing 8–16‐cell embryos with blastocysts (Melo‐Baez et al. 2020). Further, in bovine embryos produced in vitro, increased expression of miR‐24 was found to be related to reduced embryo development up to the blastocyst stage (Liang et al. 2017). In pregnancies produced by IVF in women, increased expression of miR‐125b regulates endometrial receptivity, while increased expression of miR‐1 and miR‐2 are related to a nonreceptive endometrium (Khosravizadeh et al. 2023).

Long noncoding RNAs (lncRNAs), another class of ncRNAs of over 200 nucleotides, regulate gene expression pre and posttranscription directly on gene promoters, on chromatin‐modifying enzymes, on mRNAs, and proteins, as well as acting as miRNA sponges and competing with mRNA for miRNA binding (Zhang et al. 2019). LncRNAs play a role in the development and function of most organs, including the placenta (McAninch et al. 2017). Altered expression of lncRNAs can impair the establishment of maternal–fetal communication by disrupting key processes involved in early pregnancy, such as embryo adhesion, implantation, and placentation, ultimately increasing the risk of implantation failure, pregnancy loss, and abnormal placental development (reviewed by McAninch et al. 2017). In bovine embryos, glutathione treatment led to differential expression of 4273 lncRNAs, including higher expression of lncRNA Cuff.21976.3, which was associated with decreased expression of *Klf11*, increasing cell viability and embryo development (Guo et al. 2020).

Despite studies demonstrating the importance of sEVs in maternal‐fetal communication and pregnancy success (Adam et al. 2017; Bridi et al. 2020; Buca et al. 2020; Chiarello et al. 2018; Nair and Salomon 2020), differences in the content of sEVs between AI and IVF‐derived pregnancies, and their role in supporting this dialogue, remain poorly understood (Almiñana et al. 2017; Bridi et al. 2021). Thus, in the present study, we hypothesize that the transcriptome and microtranscriptome of sEVs derived from pregnancies produced by in vitro fertilization—embryo transfer (IVF‐ET) and fixed‐time artificial insemination (FTAI) exhibit different contents, which could affect the maternal‐fetal dialogue in IVF‐ET pregnancies. We performed a global analysis of mRNAs, lncRNAs, and miRNAs in sEVs collected pre and postattachment (18 and 32 days, respectively, early stages of placentome development), in bovine pregnancies produced by FTAI and IVF‐ET, aiming to further understand maternal‐fetal communication in early pregnancy, as well as to identify differences in sEVs content in IVF‐derived pregnancies.

## 2 Methods

### 2.1 Sample Size

**Figure 1:**
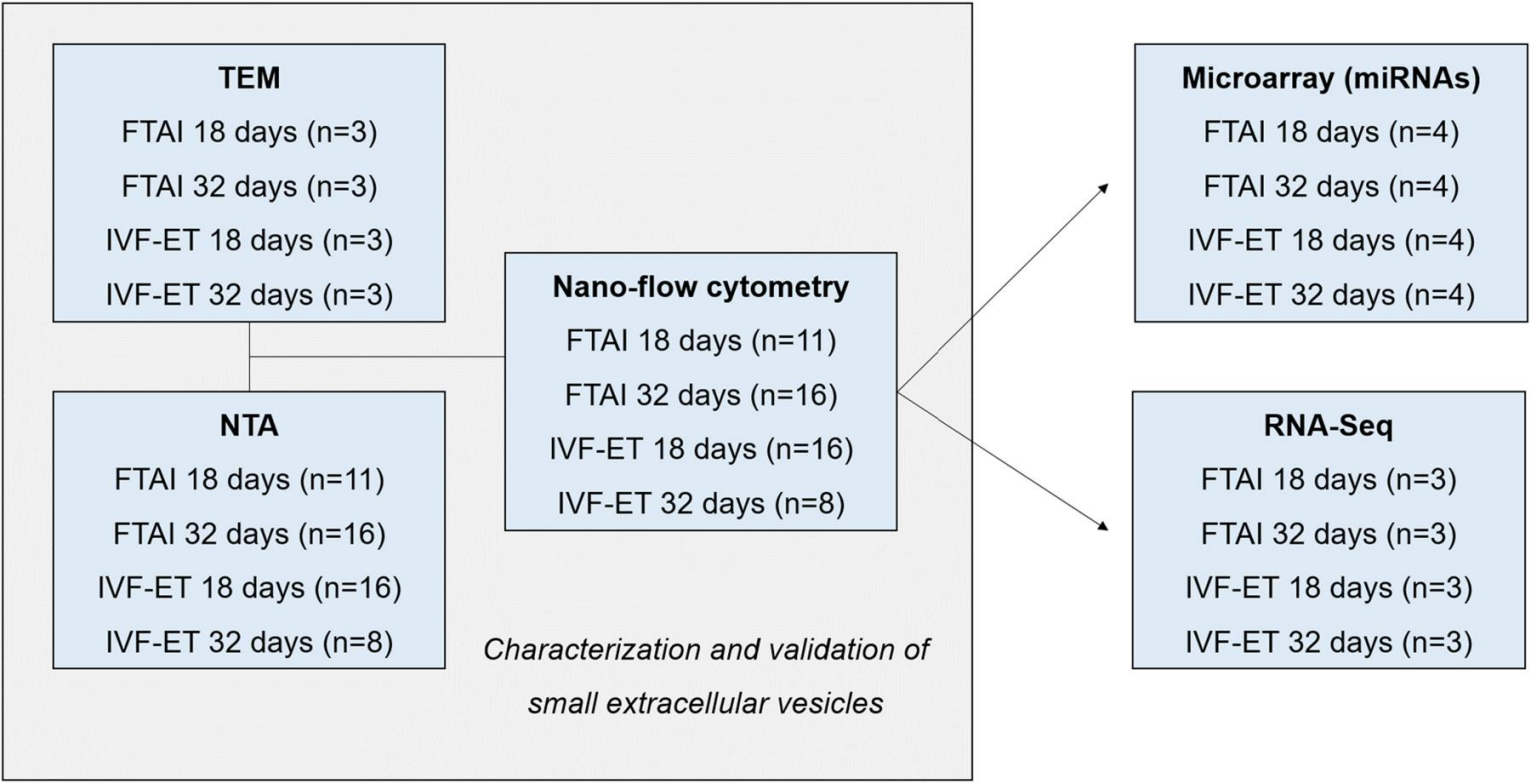
Sample distribution across analyses was as follows: Transmission electron microscopy (TEM): (*n* = 3/treatment); Nanoparticle tracking analysis (NTA): FTAI18 (*n* = 11), IVF‐ET18 (*n* = 16), FTAI32 (*n* = 16), and IVF‐ET32 (*n* = 8); Nano‐flow cytometry: FTAI18 (*n* = 5), IVF‐ET18 (*n* = 4), FTAI32 (*n* = 5), and IVF‐ET32 (*n* = 5); Microtranscriptome analysis by microarray (*n* = 4/treatment) and transcriptome analysis by RNA‐Seq (*n* = 3/treatment). Detailed description of methods is provided in the subsequent sections.

The sample size is shown in Figure 1.

### 2.2 Animals and Experimental Design

**Figure 2:**
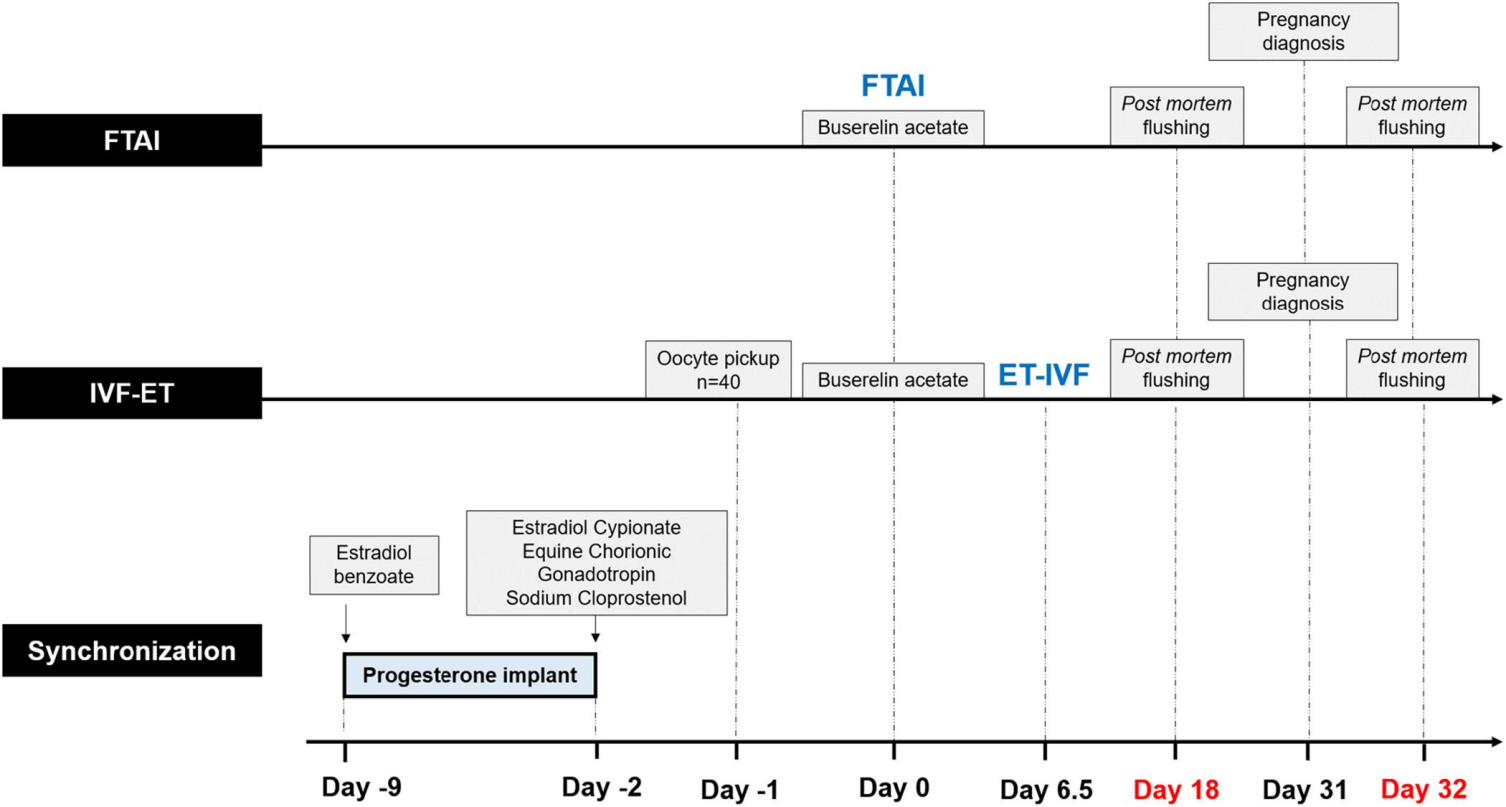
Timeline for estrous synchronization, IVF‐ET, FTAI, and collections. On Day −9, cows were selected for the estradiol benzoate (EB)‐based synchronization protocol for fixed‐time artificial insemination (FTAI) and in vitro fertilization—embryo transfer (IVF‐ET). From Day −9 to Day −2, a vaginal progesterone (P4) implant was inserted along with the administration of EB. On Day −2, cows received sodium cloprostenol (PGF2α), equine chorionic gonadotropin (eCG), and estradiol cypionate (EC), followed by progesterone implant removal. On Day −1, oocyte aspiration was performed. On Day 0, buserelin acetate (GnRH) was administered, and cows from the AI group were inseminated. At 6.5 days, embryo transfer was performed to the IVF‐ET group. Cows were slaughtered 18 and 32 days following AI (Day 0), and 12 and 26 days after embryo transfer (IVF‐ET group).

Animal handling and sample production were conducted at the Experimental Station Hildegard Georgina Von Pritzelwiltz, located in Londrina, PR, Brazil. The Animal Research Ethics Committee of the “Luiz de Queiroz” College of Agriculture (ESALQ)/University of São Paulo approved all procedures involving animals (Protocol #2018.5.1252.11.5, n° CEUA—2018–21). Nonlactating *Bos taurus indicus* (Nelore) cows with body condition scores (BCS) of 3.0 ± 0.04 on a 1–5 scale (moderate body condition) were selected for the experiment. Cows were synchronized following an estradiol benzoate (EB) timed‐AI protocol (Madureira et al. 2020). Semen used for insemination and in vitro embryo production was from a single *Bos taurus taurus* (Angus) bull, and IVF‐ET was performed as previously described by Seneda et al. (2001). On Day 0 (Figure 1), cows from the FTAI group were inseminated. On Day 6.5, embryo transfer was performed to the IVF‐ET group. Cows were slaughtered on Days 18 and 32 after FTAI (*n* = 4/day), and on Days 12 and 26 post embryo transfer (IVF‐ET group; *n* = 4/day). The days selected for uterine fluid collection represent a vital period for maternal recognition (18 days) and an ongoing process of placental attachment and development (32 days), which begins around Days 20–22 and progresses throughout gestation. Experimental timeline is depicted in Figure 2.

### 2.3 Pregnancy Diagnosis and Tissue Processing

After slaughter, at 18 and 32 days of pregnancy, the entire reproductive tract (uterus, cervix, and both ovaries) was immediately removed and placed in plastic bags, sealed, and kept on ice until processing. Ipsilateral and contralateral uterine horns were identified postmortem at 18 and 32 days, and the reproductive tract was trimmed to remove excess tissue, facilitating uterine horn flushing. On Days 18 and 32, each horn (the ipsilateral horn followed by the contralateral horn) was flushed using sterile saline solution, with great care to avoid tissue rupturing. At Day 32, prior to flushing, all fetal/placental content was carefully removed through a cut in the cervix, checking for the integrity of all membranes in order to ascertain that we were flushing uterine content and not allantoic or amniotic fluid. Flushing content was then transferred to a sterile petri dish (100 mm) for the subsequent isolation of sEVs. Day 18, the pregnancy was confirmed by the presence of an embryo in the uterine flushes. For Day 32, pregnancy diagnosis, performed by ultrasonography to detect the heartbeat, occurred on Day 31, 1 day prior to slaughter. Samples of uterine fluid from 16 cows were selected, 4 samples/treatment group on Day 18 and 4 samples/treatment group on Day 32.

### 2.4 sEV Isolation

Samples of uterine fluid were centrifuged three times to remove live cells, cell debris, and large microvesicles (300 × g for 10 min, 2000 × g for 10 min, and 16,500 × g for 30 min). The supernatant was then passed through 0.22 μm sterile syringe filters (Millipore, polyethersulfone membrane), a step commonly used to eliminate particles larger than 200 nm without significantly compromising sEV yield. Filtered samples were transferred to 3.5 mm‐thick‐wall polycarbonate tubes, and phosphate‐buffered saline (PBS) was added to adjust the volume to 20 mL. Samples were then ultracentrifuged using an Optima XE‐90 ultracentrifuge at 34,100 rpm (∼100,000 × g) for 70 min at 4°C to sediment the sEVs. The supernatant was removed, and pellets were washed with 10 mL PBS and centrifuged again under the same conditions. Final pellets, enriched in sEVs, were resuspended in 1 × PBS without calcium (Ca^2+^) or magnesium (Mg^2+^) and stored (De Bem et al. 2024). Although ultracentrifugation may affect vesicle integrity and RNA recovery, it remains one of the most widely used methods for sEV isolation due to its ability to process large sample volumes without the use of chemical precipitation reagents and to provide reproducible enrichment of vesicles suitable for downstream RNA analyses (Konoshenko et al. 2018).

### 2.5 Transmission Electron Microscopy of sEVs

Transmission electronic microscopy (TEM) was employed for morphological analysis (shape and size) of sEVs uterine fluid from FTAI and IVF‐ET pregnancies at Days 18 and 32 (*n* = 3/treatment). Pellets of sEVs/microvesicles were fixed in a solution of 2.5% glutaraldehyde, 5% saccharose, and 0.1 M sodium cacodylate buffer, pH 7.4, for 30 min at room temperature. Then, they were postfixed in 1% osmium tetroxide and 0.1 M sodium cacodylate buffer solution and centrifuged at 4°C. Osmicated pellets were dehydrated in an ethanol gradient, washed in propylene oxide, and soaked in Poly/Bed 812 resin (Polysciences Inc). Sections of 80 nm were obtained, mounted on 300‐mesh screens, and stained with uranyl acetate and lead citrate (da Silveira et al. 2012).

### 2.6 Nanoparticle Tracking Analysis (NTA) of sEVs

NTA was employed to determine the size distribution and concentration of sEVs. Samples containing sEVs were analyzed using the NanoSight NS300 (Malvem) equipment and the NanoSight NTA Software v3.1, and for equipment calibration, beads of 50, 100, and 200 nm were used. Samples containing sEVs, diluted in 100 μL, were classified by size and concentration for FTAI at 18 days (*n* = 11) and 32 days (*n* = 16), and for IVF‐ET at 18 days (*n* = 16) and 32 days (*n* = 8). Classification was performed using five 30‐second videos (camera level 14) at 37°C.

### 2.7 Statistical Analysis of sEVs

All data are presented as mean ± standard error. The Kolmogorov–Smirnov test was applied to evaluate data distribution. As concentration data did not present normal distribution, they were transformed using the base‐10 logarithm, and normality was reassessed following transformation. To analyze the effect of time (Days 18 x 32), an unpaired *t*‐test with Welch's correction and one‐way ANOVA were applied. Probability values ≤ 0.05 were considered statistically significant. All calculations and analyses were performed using GraphPad Prism v.5.0. Samples were separated into aliquots of 50 μL and stored at −80°C for the extraction of total RNA from extracellular vesicles.

### 2.8 Characterization of sEVs by Nano‐Flow Cytometry

For the characterization of sEVs by nano‐flow cytometry, a total of 60 µL was used for cytometry analysis and 10 µL for Nanosight NS300 (Malvem). Samples were pooled from each different experimental group, and isolated samples were incubated with fluorescence‐conjugated antibodies for CD81‐FITC (1:10 v/v), Syntenin‐PE (1:40 v/v), Alix‐PE (1:40 v/v), and CD63‐FITC (1:10 v/v) for 2 h at room temperature on a shaker. To detect intracellular proteins such as Alix and Syntenin, membrane permeabilization was performed prior to antibody incubation using 0.001% Triton (1:1 v/v) for 15 min at room temperature. Additionally, before incubation with the sample, antibodies were centrifuged at 20,000 × g for 30 min at 4°C to sediment any particles that could interfere with the analysis. Samples were diluted in 200 µL of PBS filtered three times and analyzed using a CytoFLEX instrument (Beckman Coulter, USA). Cytometer was configured to detect nanoparticles according to the fluorophore wavelength conjugated to the antibody. Gain and threshold values were adjusted based on the recommendations for size beads (100–300 nm) and the fluorescence of interest. Gating was determined based on the negative control for each antibody (PBS + antibody). As a negative control, the endoplasmic reticulum marker Calnexin (1:50 v/v) was used to detect any contamination in the isolate. For standardization, a reading of 50 µL per sample was performed at a low flow rate (10 µL/min) (Figure S1).

### 2.9 RNA Extraction of sEVs

Total RNA from sEVs was extracted using the mirVana™ miRNA Isolation Kit (Thermo Fisher Scientific), following the manufacturer's instructions. RNA concentration and purity were assessed using a NanoDrop 2000 spectrophotometer (Thermo Fisher Scientific). RNA integrity was evaluated using an Agilent 2100 Bioanalyzer prior to library preparation, and only samples with RNA Integrity Number (RIN) values above 7.0 were used for subsequent analysis.

### 2.10 RNA‐Sequencing (RNA‐Seq) Library Preparation

Samples of total RNA from 12 cows undergoing FTAI and IVF‐ET at 18 and 32 days were selected for RNA sequencing library preparation. Samples were selected based on RNA quality to maximize library preparation success and sequencing performance. Total RNA sequencing libraries were constructed from 150 ng of RNA/sample. Libraries were prepared using the Zymo‐Seq RiboFree total RNA library Prep Kit (Zymo Research Co.), following the manufacturer's protocol. Libraries were sequenced using the Illumina NovaSeq to a sequence depth of a minimum of 30 million read pairs/sample (150 bp paired‐end).

### 2.11 Bioinformatic Analysis of RNA‐Seq Data

RNA‐Seq data were obtained as FASTQ files for 12 samples (*n* = 3/treatment group), and quality control was carried out using FastQC v.0.11.9 (http://www.bioinformatics.babraham.ac.uk/projects/fastqc/). All sequences with adapter and low‐quality were trimmed using Trim Galore! v.0.6.6 (https://www.bioinformatics.babraham.ac.uk/projects/trim_galore/). Trimmed reads were aligned to the reference genome (*Bos taurus*) assembly ARS‐UCD1.2 using STAR v.2.6.1 d (Dobin et al. 2013). Reads overlapping with exons were assigned to genes using featureCounts v.2.0.1 for expression quantification (Liao et al. 2014). Quality control of all steps was visualized using MultiQC v.1.9 (Ewels et al. 2016).

### 2.12 Identification of Differentially Expressed (DE) Transcripts and Co‐Expression Analysis Between mRNAs and lncRNAs

For the identification of DE genes from RNA‐Seq data, we employed the DESeq. 2 package (v1.38.3) in R (v4.3.0), within the Bioconductor framework (v3.17) (Love et al. 2014). Raw counts were filtered to retain transcripts with a minimum count of ≥ 10 in at least half of the samples within one of the groups. Normalization was performed using DESeq. 2, a method based on the estimation of size factors. Differential expression analysis was then conducted using the Wald test implemented in DESeq. 2. After the identification of DE genes |Log_2_(FC)| ≥ 1, FDR ≤ 0.01) of 12 samples, biotype classification was performed using the BioMart tool (Martin et al. 2023), and transcripts of “lncRNA” and “protein_coding” types were selected. Next, co‐expression analysis of DE mRNAs and lncRNAs was performed using Pearson's correlation coefficient, with the tidyverse package (v2.0.0) in R (v4.3.0). LncRNA‐mRNA interactions were classified as significant if the Pearson correlation coefficient |*r*| ≥ 0.99 and the *p*‐value was corrected for multiple hypotheses using the Bonferroni correction method, with a significance threshold of < 0.1.

The potential of ligation between lncRNAs and putative target mRNAs was analyzed using the LncTar tool (Li et al. 2014), based on the estimation of normalized free energy (ndG ≤ −0.10) for each interaction. LncRNAs were classified with *cis* action potential using the Classifier Module of Flexible Extraction of long noncoding RNAs (FEELnc) tool (Wucher et al. 2017). *Cis* acting lncRNAs are predicted to influence nearby partner genes, thus a correlated lncRNA falling within a window of 100 kbps upstream or downstream of its target mRNA, was classified as *cis‐*acting. Correlated pairs with binding potential (ndG ≤ −0.10) outside of the 100 kbps window were considered as potential *trans‐*acting.

### 2.13 Gene Set Enrichment Analysis (GESA)

All expressed mRNAs identified by RNA‐Seq were subjected to orthology analysis against human genes using Biomart (Ensembl). Human orthologs were used for functional enrichment analyses in order to take advantage of robust and comprehensive pathway annotation available for human genes in the KEGG and MSigDB databases. Only mRNAs with orthology confidence scores = 1 (high confidence) were selected for further analysis. GSEA was performed for all treatment groups on the GSEA software v.4.3.2 (Subramanian et al. 2005) using normalized counts generated in DESeq. 2 tool and filtered to retain transcripts with a minimum count of ≥ 10 in at least half of the samples within one of the groups. GSEA was performed using the following parameters: gene set database = h.all.v2023.1.Hs.symbols.gmt (Hallmark) and c2.cp.kegg.v2023.1.Hs.symbols.gmt (KEGG); number of permutations = 1.000; collapse = true; permutation type = gene_set; chip plataform = Human_ensembl_gene_ID; enrichment statistic = weighted, and metric for ranking genes = Signal2Noise. Molecular signatures database v2023 (MSigDB) was used to identify pathways. Gene sets were considered significantly enriched at FDR *q* < 0.005 (Subramanian et al. 2005).

### 2.14 Microtranscriptome Analysis by Microarray

Total RNA from 16 pregnancies (*n* = 4/treatment group) were used to microarray analysis. Analysis was conducted using 250 ng of RNA/sample using the Affymetrix® miRNA 4.1 Array Strip, with 30,424 mature miRNAs to different organisms, following the manufacturer's protocol. This essay allows for global microtranscriptome analysis of several species, including *Bos taurus*. Briefly, four samples per treatment were marked, hybridized, washed, and scanned in the GeneAtlas (Affymetrix®) equipment. Results obtained were normalized by the RMA + DABG method. Differential expression analysis was performed to contrast techniques: IVF‐ET18 x FTAI18 and IVF‐ET32 x FTAI32 (FC ± 1.5 and *p*‐adj ≤ 0.05); and to contrast pregnancy stages within each treatment: FTAI32 x FTAI18 and IVF‐ET32 x IVF‐ET18 (FC ± 1.5 and *p*‐adj ≤ 0.05). ANOVA with the eBayes correction method was employed for analysis using the Transcriptome Analysis Console 4.0 (Thermo Fisher Scientific Co.) software.

### 2.15 Prediction of Target mRNAs of DE miRNAs

Target prediction for DE miRNAs for all contrasts was performed using miRmap (Vejnar and Zdobnov 2012). We only considered as possible targets, mRNAs with a score above 90%, based on site accessibility energy (∆G open), probability, and phyloP conservation, following the predictor specifications. Predicted targets were compared with DE mRNAs (RNA‐Seq), and only predicted targets that were DE, within the same contrast, were considered as final targets.

### 2.16 Pathway Enrichment Analysis of Target mRNAs of DE miRNAs

Target mRNAs of DE miRNAs were subjected to orthology analysis with human transcripts. Subsequently, pathway enrichment analysis was performed using ClueGO, based on KEGG pathways. The *p*‐values (*p* ≤ 0.05) were corrected for multiple testing using the Benjamini–Hochberg false discovery rate (FDR) method.

### 2.17 Analysis of DE lncRNA–miRNA–mRNA Interactions

All DE lncRNAs, mRNAs, and miRNAs were used to perform a competing endogenous RNA (ceRNA) analysis. The miRanda algorithm (https://github.com/hacktrackgnulinux/miranda) was used to predict lncRNA–miRNA interactions with a score of ≥ 140 and an energy cutoff of ≤ −10 kcal/mol. Subsequently, the SPINNAKER tool (https://github.com/sportingCode/SPINNAKER) was employed to identify potential lncRNA–miRNA–mRNA regulatory triplets based on Pearson correlation analysis and expression patterns. Only interactions showing the expected ceRNA correlation pattern: lncRNA–miRNA (*r* < 0), miRNA–mRNA (*r* < 0), and lncRNA–mRNA (*r* > 0) and with a Benjamini–Hochberg adjusted *p*‐value (FDR) ≤ 0.05 were considered significant.

## 3 Results

### 3.1 Characterization of sEVs

All samples exhibited uniform particles with a nearly regular spherical shape (Figure S2). On contrasting days (18 × 32) within each reproductive technique, sEVs from Day 32 were smaller than those from Day 18 in the IVF‐ET (123.83 ± 0.78 nm and 116.30 ± 1.73 nm; *p* = 0.003). Regarding sEVs concentration, a higher concentration was observed in the Days 18–32 of pregnancy in FTAI and IVF‐ET (*p* = 0.03; Figure S3). Nano‐flow cytometry was also used to quantify the number of events per µL in the different experimental groups (Table 1).

**Table 1:** Number of events per µL detected by nano‐flow cytometry in the different experimental groups using various extracellular vesicle markers. CD63 and CD81 are membrane markers; Alix and Syntenin are intracellular protein markers; and Calnexin serves as a negative control marker. Samples were obtained from artificially inseminated and fertilized cows on Day 18 (FTAI18 and IVF‐ET18), and artificially inseminated and fertilized cows on Day 32 (FTAI32 and IVF‐ET32). The positive control group consisted of PBS solution supplemented with atc.

Samples	CD63	CD81	Alix	Sintenix	Caldexin
PBS + atc	1.62	4.61	40.33	3.9	0.96
IVF‐ET18	52.02	34.3	567.31	63.93	2.24
FTAI18	23.82	32.88	329.64	20.76	3.18
IVF‐ET32	56.85	32.8	532.6	2.62	2.16
FTAI32	87.5	9.52	573.15	6.32	2

### 3.2 Differential Expression of mRNAs

**Figure 3:**
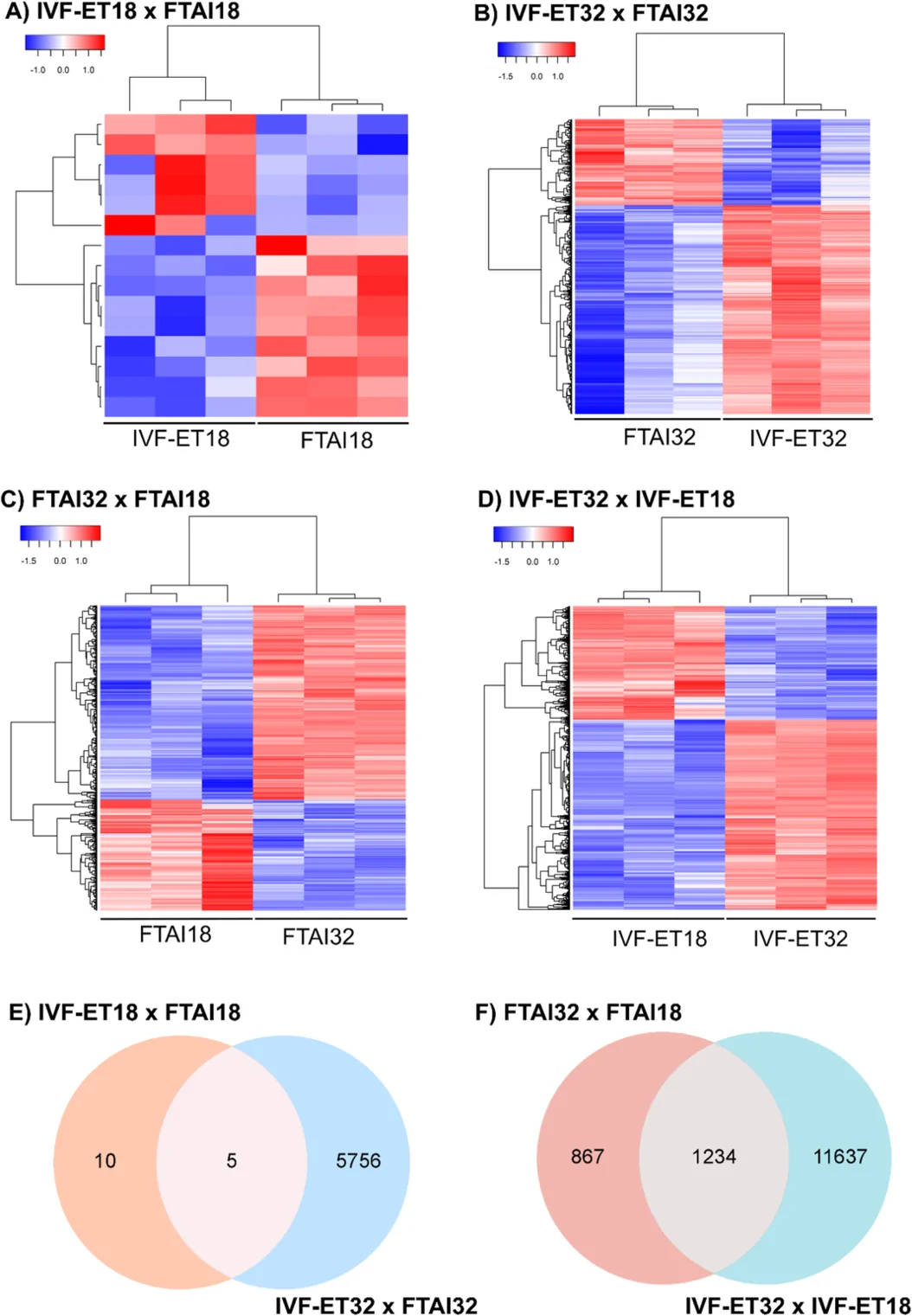
Heatmap for DE mRNAs (|Log_2_(FC)| ≥ 1 and FDR ≤ 0.01). Heatmap for DE mRNAs (|Log_2_(FC)| ≥ 1 and FDR ≤ 0.01) at (A) 18 days in IVF‐ET and FTAI gestations, (B) 32 days in IVF‐ET and FTAI gestations, (C) 32 and 18 days in FTAI gestations, and (D) 32 and 18 days in IVF‐ET gestations. Heatmaps represent hierarchical clustering (complete linkage method with Euclidean distance) of DE mRNA expression according to the color scale in z‐score, where red indicates up‐regulated mRNAs and blue indicates down‐regulated mRNAs. White represents no significant change in expression. Venn diagram of DE mRNAs (|Log_2_(FC)| ≥ 1 and FDR ≤ 0.01) at (E) 18 days in IVF‐ET and FTAI gestations and 32 days in IVF‐ET and FTAI gestations, and (F) 32 and 18 days in FTAI gestations and 32 and 18 days in IVF‐ET gestations.

In order to further understand maternal‐fetal communication mediated by sEVs, at 18 (pre‐adherence) and 32 days (postadherence) of pregnancies produced by IVF‐ET and FTAI, transcriptome analysis was performed. All expressed transcripts were filtered |Log_2_(FC)| ≥ 1 and FDR ≤ 0.01) for identification of DE transcripts. When contrasting techniques, 6 up‐ and 9 down‐regulated mRNAs were found in IVF‐ET18 x FTAI18, whereas 4088 up‐ and 1673 down‐regulated mRNAs were observed in IVF‐ET32 x FTAI32. When comparing gestational days (32 × 18) within each technique, 1339 mRNAs were up‐regulated, and 762 were down‐regulated in FTAI32 x FTAI18, whereas striking 8053 up‐ and 4818 down‐regulated mRNAs were found in IVF‐ET32 x IVF‐ET18. Normalized counts and differential expression results are provided in Table S1. Differential expression of DE mRNAs for all contrasts are presented in heatmaps (Figure 3A–D). Next, differences between contrasts within gestational day (Figure 3F) and within reproductive technique (Figure 3E) are shown.

Overall, we observed that differences in mRNA content between reproductive techniques are much more pronounced at Day 32 of gestation (Figure 2). Also, within each technique, the changes in transcript expression, expected to occur as gestation progresses (32 x 18 days), is seven times higher in IVF‐ET gestations (Figure 2) when compared to FTAI.

### 3.3 GSEA

All expressed mRNAs that had ortholous in the human genome were subjected to GSEA analysis (87.61% in IVF‐ET18 x FTAI18; 81.07% in IVF‐ET32 x FTAI32; 86.18% in FTAI32 x FTAI18, and 81.39% in IVF‐ET32 x IVF‐ET18). Gene sets were considered significantly enriched when FDR *q*‐value (FDR *q*‐value) was < 0.005. Analysis was carried out for all four contrasts (Table S2). GSEA results revealed that in the IVF‐ET18 x FTAI18 contrast, 22 gene sets were up‐regulated in IVF‐ET18 (*q*‐value < 0.005), such as ROS and protein export pathways. A total of 24 gene sets in IVF‐ET32 x FTAI32 were up‐regulated in IVF‐ET32 (*q*‐value < 0.005). Among these, a number of pathways related to immunological regulation are of particular relevance to early pregnancy. When we run the analysis within each technique, to evaluate pregnancy progression, a total of 30 gene sets in IVF‐ET32 x IVF‐ET18 were up‐regulated in IVF‐ET32 (*q*‐value < 0.005). Among these, immune regulation pathways and ECM receptor interaction are highly relevant for our study (Figure S4). Only two gene sets in FTAI32 x FTAI18 were up‐regulated in FTAI32 (*q*‐value < 0.005), including the Hallmark Hypoxia, which is of particular interest (Table S2).

### 3.4 Differential Expression of lncRNAs

**Figure 4:**
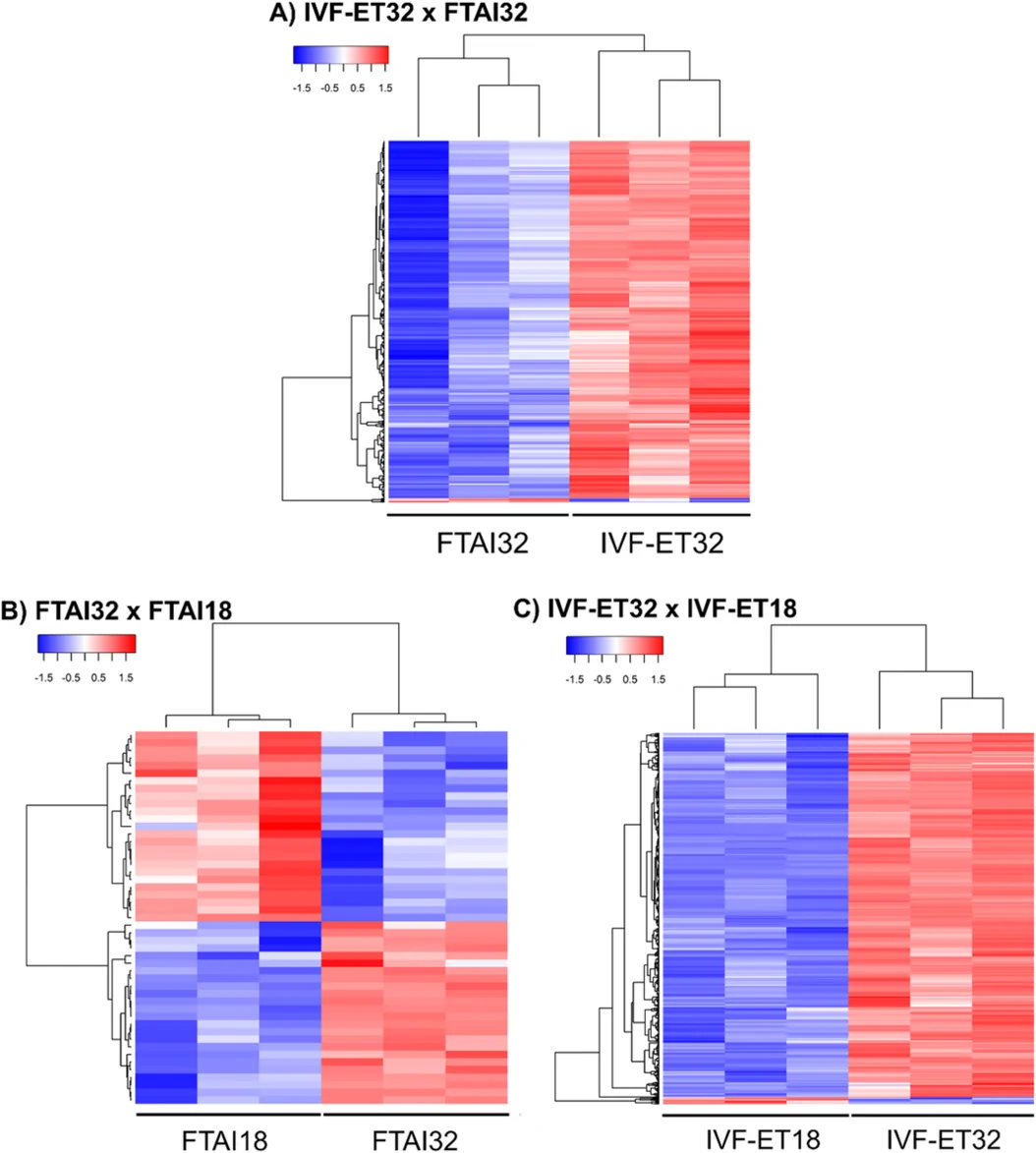
Heatmap for DE lncRNAs (|Log_2_(FC)| ≥ 1 and FDR ≤ 0.01). Heatmap for DE lncRNAs (|Log_2_(FC)| ≥ 1 and FDR ≤ 0.01) at (A) 32 days in IVF‐ET and FTAI gestations, (B) 32 and 18 days in AI gestations, and (C) 32 and 18 days in IVF‐ET gestations. Heatmaps represent hierarchical clustering (complete linkage method with Euclidean distance) of DE lncRNA expression according to the color scale in z‐score, where red indicates up‐regulated mRNAs and blue indicates down‐regulated mRNAs. White represents no significant change in expression.

Following biotype analysis of transcripts obtained from RNA‐Seq, 530 lncRNAs were detected in the IVF‐ET18 x FTAI18 contrast, 1308 in IVF‐ET32 x FTAI32, 782 in FTAI32 x FTAI18, and 1303 in IVF‐ET32 x IVF‐ET18. Among these, no DE lncRNAs were identified in IVF‐ET18 x FTAI18, whereas 398 were up‐ and 5 down‐regulated in IVF‐ET32 x FTAI32, 24 up‐ and 25 down‐regulated in FTAI32 x FTAI18, and 1081 up‐ and 21 down‐regulated in IVF‐ET32 x IVF‐ET18 (Table S1). LncRNAs DE between IVF‐ET and FTAI at 32 days are presented in Figure 4A, and a clear predominance of up‐regulation of lncRNAs is found in the IVF‐ET group. Our results also show that the number of lncRNAs up‐ and down‐regulated during the evolution of pregnancy, between Days 18 and 32, is balanced in AI pregnancies (FTAI32 x FTAI18; Figure 4B), whereas the vast majority of lncRNAs are up‐regulated as gestation progresses in the IVF‐ET group (Figure 4C).

Following Pearson's correlation analysis, 54 (IVF‐ET32 x FTAI32), 30 (FTAI32 x FTAI18), and 241 (IVF‐ET32 x IVF‐ET18) significant interactions were found between DE lncRNAs and DE mRNAs (|*r*| ≥ 0.99; Bonferroni‐adjusted *p*‐value < 0.1) (Table S3). Using the LncTar tool, we found that 7 (IVF‐ET32 x FTAI32), 5 (FTAI32 x FTAI18), and 24 (IVF‐ET32 x IVF‐ET18) of those lncRNA‐mRNA interactions had *trans*‐acting binding potential (ndG < −0.10) (Table S4). According to their genomic location (within a 100 kb window), no lncRNA–mRNA interactions between neighboring genes (*cis*‐acting) were identified in the IVF‐ET32 x IVF‐ET18, FTAI32 x FTAI18, and IVF‐ET32 x FTAI32.

**Figure 5:**
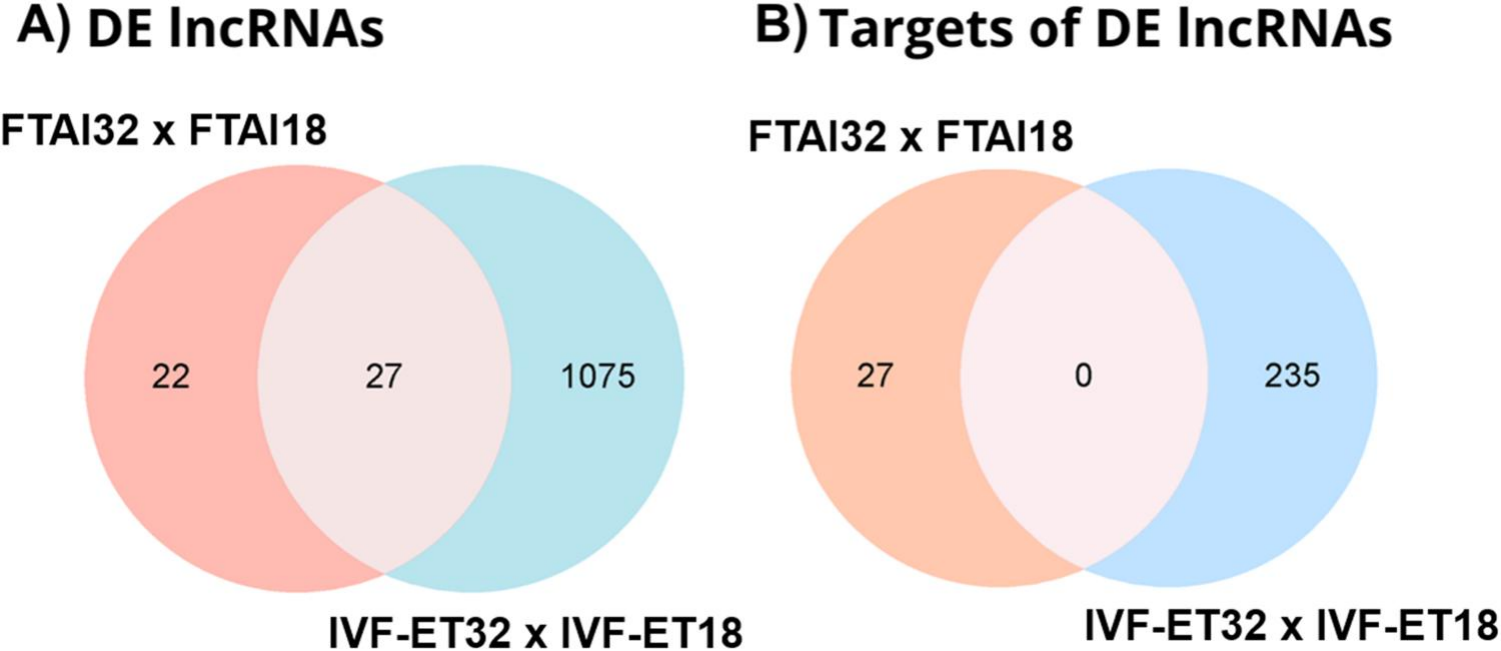
Venn diagram of DE lncRNAs (|Log_2_(FC)| ≥ 1 and FDR ≤ 0.01) and target mRNAs of DE lncRNAs. Venn diagram of DE lncRNAs (|Log_2_(FC)| ≥ 1 and FDR ≤ 0.01) and target mRNAs of DE lncRNAs at (A) DE lncRNAs identified at 18 and 32 days in FTAI and IVF‐ET pregnancies and (B) Venn diagram representing the predicted DE mRNAs, targets of DE lncRNAs, identified based on Pearson correlation coefficient (|*r*| ≥ 0.99 and Bonferroni‐adjusted *p*‐value < 0.1) at 18 and 32 days in FTAI and IVF‐ET pregnancies. Overlapping regions indicate mRNAs that are commonly targeted by DE lncRNAs across different groups, while nonoverlapping regions represent unique targets for each condition.

Next, we compared DE lncRNAs between gestational days for each reproductive technique. Only 27 DE lncRNAs were found to be shared across gestational progression in both techniques (Figure 5A). However, no common putative target mRNAs of DE lncRNAs were identified between the groups (Figure 5B). The vast majority of DEs were exclusive to the IVF‐ET contrast, both for lncRNAs and correlated target mRNAs.

### 3.5 Differential Expression of miRNAs

**Figure 6:**
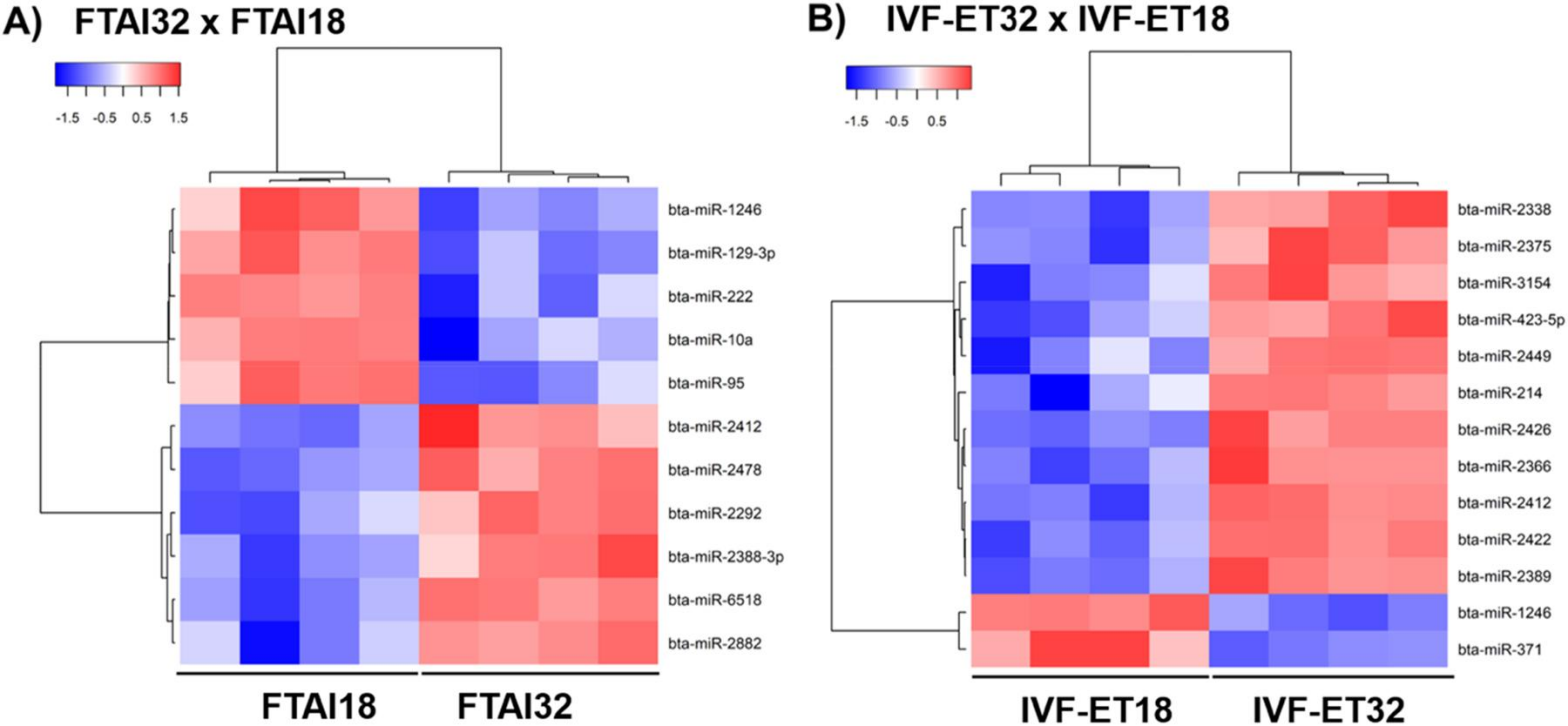
Heatmap for DE miRNAs (FC ± 1.5 and *p*‐adj ≤ 0.05). Heatmap for DE miRNAs at (A) 32 and 18 days in AI gestation (FC ± 1.5 and *p*‐adj ≤ 0.05) and (B) 32 and 18 days in IVF‐ET gestation (FC ± 1.5 and *p*‐adj ≤ 0.05). Heatmaps represent hierarchical clustering (complete linkage method with Euclidean distance) of DE miRNA expression according to the color scale in z‐score, where red indicates up‐regulated mRNAs and blue indicates down‐regulated mRNAs. White represents no significant change in expression.

Through microtranscriptome analysis within each technique, we identified 6 up‐ and 5 down‐regulated miRNAs in AI32 x AI18 and 11 up‐ and 2 down‐regulated miRNAs in IVF‐ET32 x IVF‐ET18 (Table S5). No DE miRNAs were found between techniques, in IVF‐ET18 x FTAI18 and IVF‐ET32 x FTAI32. Heatmaps of DE miRNAs show clustering by gestational stage (Figure 6). Observing the differential pattern of expression of miRNAs between gestational days, within each technique, only bta‐miR‐2412 and bta‐miR‐1246 had similar temporal patterns in both techniques (Figure 6A,B). Similar to what was observed for lncRNAs, the number of miRNAs up‐ and down‐regulated, between Days 18 and 32, is balanced in FTAI pregnancies (Figure 6A), whereas the majority of miRNAs are down‐regulated as gestation progresses in the IVF‐ET group (Figure 6B), opposite to what we observe for lncRNAs (Figure 4C).

Among the DE miRNAs found in each treatment and day, 81% of DE miRNAs in FTAI32 x FTAI18 and 84% of DE miRNAs in IVF‐ET32 x IVF‐ET18 were specific to FTAI or IVF‐ET. All predicted target mRNAs were contrasted with DE transcripts (RNA‐Seq results), and only those that were also confirmed as DE |Log_2_(FC)| ≥ 1 and FDR ≤ 0.01 proceeded as targets for pathway enrichment analysis. Therefore, the identified miRNA–mRNA interactions should be considered predicted associations rather than experimentally validated regulatory relationships. Following target prediction, 106 DE mRNAs were found as targets to 11 DE miRNAs (AI32 x AI18), 1128 were found as targets to 12 out of 13 DE miRNAs (IVF‐ET32 x IVF‐ET18). All predicted targets, regardless of their status on the RNA‐Seq, are shown in Table S6.

### 3.6 Analysis of DE lncRNA–miRNA–mRNA interactions

To explore potential competing endogenous RNA (ceRNA) interactions, DE lncRNAs, mRNAs, and miRNAs were integrated. Unlike other analyses in this study, where orthology with *Homo sapiens* was applied for functional annotation, ceRNA analysis was performed using bovine DE transcripts directly. LncRNA–miRNA interactions were predicted using miRanda (Table S7), and ceRNA triplets were identified with SPINNAKER based on expression correlation and statistical significance (FDR ≤ 0.05). Through these analyses, we identified 50 lncRNA–miRNA–mRNA interactions in FTAI32 × FTAI18 and 1321 interactions in IVF‐ET32 × IVF‐ET18 (Table S8). These interactions showed the expected regulatory pattern, where upregulated lncRNAs were associated with downregulated mRNAs and upregulated miRNAs; conversely, downregulated lncRNAs were associated with upregulated mRNAs and downregulated miRNAs. These findings suggest potential ceRNA‐mediated regulation but should be interpreted cautiously, as they are based on predictive analyses.

### 3.7 Pathway Enrichment Analysis of Putative Target mRNAs of DE miRNAs

**Figure 7:**
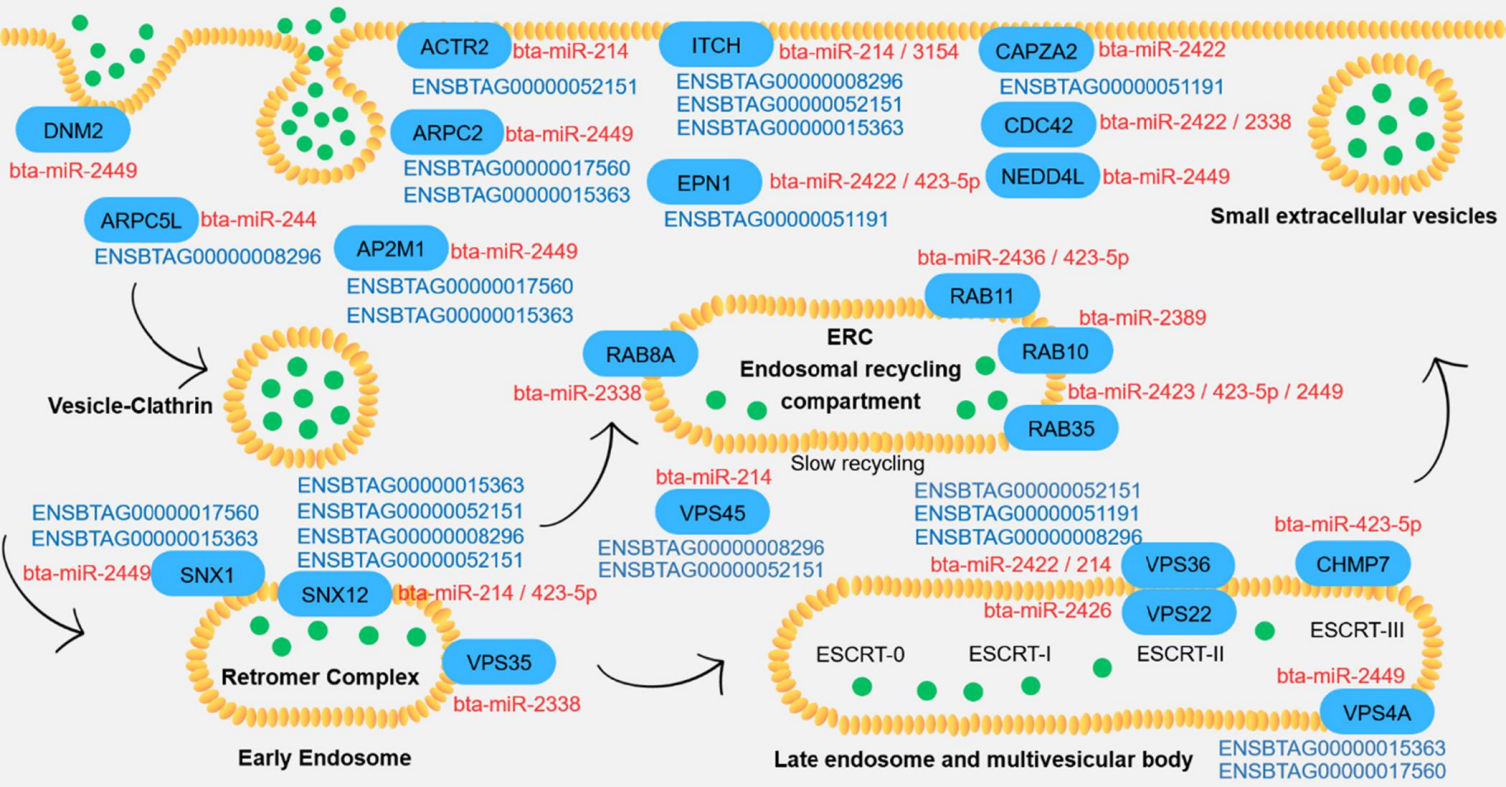
Endocytosis pathway of targets of upregulated DE miRNAs (FC ± 1.5 and *p*‐adj ≤ 0.05) in IVF‐ET32 x IVF‐ET18. Legend: Blue circle represents down‐regulated DE mRNAs (|Log2(FC)| ≥ 1 and FDR ≤ 0.01). Red font represents upregulated DE miRNAs and blue represents down‐regulated DE lncRNAs (|Log2(FC)| ≥ 1 and FDR ≤ 0.01). Endocytosis enables the internalization of proteins via clathrin‐coated vesicles. *ITCH* and *NEDD4L* tag membrane proteins for uptake, while *AP2M1* and *EPN1* recruit clathrin and induce membrane curvature. *DNM2* mediates vesicle scission, and actin polymerization (*ARPC2*, *ARPC5L*, *ACTR2*, *CAPZA2*, *CDC42*) supports trafficking. Internalized cargo is sorted in early endosomes (*SNX1*, *SNX12, VPS35*) toward recycling, retrograde, or degradative pathways. Recycling involves *RAB35*, *RAB8A*, *RAB10*, *RAB11*, and *VPS45*. Maturation into multivesicular bodies (MVBs) relies on the ESCRT complex (*VPS36*, *VPS22*, *CHMP7*, *VPS4A*) for intraluminal vesicle formation.

Pathway enrichment analysis was performed for DE target mRNAs of DE miRNAs (FTAI32 x FTAI18 and IVF‐ET32 x IVF‐ET18) to identify overrepresented biological functions in each group. Enrichment analysis, separated by expression pattern (up‐ and down‐regulated), was conducted using Cytoscape (ClueGO). Among the 54 up‐regulated mRNAs targeted by the down‐regulated miRNAs in AI32 x AI18, only the prostate cancer pathway was enriched, and it included just three DE mRNAs: *PLAU* (*p*‐adj < 0.003), *RB1* (*p*‐adj < 0.0005), and *TCF7* (*p*‐adj < 5.58 × 10^−13^). However, among the 1114 down‐regulated mRNAs targeted by the up‐regulated miRNAs in IVF‐ET32 x IVF‐ET18, 51 pathways were identified, with particular interest in the endocytosis pathway (Figure 7), which is highly relevant to our study model (Table S9).

Through ceRNA analysis, three lncRNA–miRNA–mRNA interactions, previously associated with the prostate cancer pathway were identified: lncRNA ENSBTAG00000051012 (|Log2(FC)| ≥ + 4.64) ‐ bta‐miR‐129‐3p (FC = −9.44 FDR = 0.01)—mRNA *PLAU* (|Log2(FC)| ≥ + 2.04); lncRNA ENSBTAG00000052722 (|Log2(FC)| ≥ + 2.41)—bta‐miR‐129‐3p (FC = −9.44 FDR = 0.01)—mRNA RB1 (|Log2(FC)| ≥ + 1.44); lncRNA ENSBTAG00000052722 (|Log2(FC)| ≥ + 2.41)— bta‐miR‐129‐3p (FC = −9.44 FDR = 0.01)—mRNA *TCF7* (|Log2(FC)| ≥ + 3.02). Interestingly, 12 mRNAs within the endocytosis pathway were found to be involved in ceRNA interactions, being targeted by both lncRNAs and miRNAs, suggesting potential regulatory roles of lncRNAs through miRNA sponging in this pathway (Figure 7).

## 4 Discussion

sEVs are secreted by both maternal and fetal/conceptus‐derived tissues during physiological and pathological conditions that can mediate maternal‐fetal communication (Czernek and Düchler 2020). Studies indicate that gestational success is dependent upon efficient maternal‐fetal communication (Giacomini et al. 2017; Nakamura et al. 2020; Saadeldin et al. 2015), and failures in IVF may be related not only to an incorrect establishment of this dialogue, but also to its absence, as initial embryo development in an in vitro environment takes place without exposure to maternal factors (Idelevich and Vilella 2020). Focusing on maternal‐fetal communication mediated by sEVs, differences in the vesicle content could be associated with gestational success in IVF‐ET and FTAI.

In cows, sEVs released from embryos produced in vitro and in vivo on Day 7 (blastocyst stage) were previously evaluated. Size and concentration of sEVs were higher in embryos produced in vivo (Aguilera et al. 2023). We failed to find differences in sEV sizes on Day 18 between FTAI and IVF‐ET. These differences may be due to the developmental stage and also to the fact that our vesicles were separated straight from the uterine fluid and can, theferore, be of either embryonic and uterine origin. When analyzing the blood of pregnant and nonpregnant mice, the size and concentration of sEVs were assessed. It was observed that the concentration of these vesicles increased as the gestational period progressed, while the size remained similar (Sheller‐Miller et al. 2019). In line with our findings, the concentration of sEVs also increased over time (Days 18–32) in both IVF‐ET and FTAI groups. However, the size of the sEVs was smaller in IVF‐ET32 compared to IVF‐ET18.

As gestation progresses, differences in vesicle content are expected to occur, however, when we compared days of gestation within each technique, variation in vesicle content of coding and noncoding transcripts was seven times higher in IVF‐ET32 x IVF‐ET18 than in FTAI32 x FTAI18. Studies investigating temporal change in transcripts derived from different reproductive techniques are scarce, notwithstanding, Clemente et al. (2011) reported that twice as many transcripts were DE between ay 7 and Day 13 embryos (Clemente et al. 2011), when comparing in vitro with in vivo produced embryos. These results suggest that temporal changes in transcripts can be influenced by reproductive technique. However, other factors beyond reproductive techniques may also contribute to the increased transcriptomic variation observed in IVF‐ET comparisons, including the cellular origin of extracellular vesicles (maternal vs*.* fetal/conceptus‐derived), the physiological status of the cow, differences in the uterine microenvironment, and intrinsic variability in embryo quality (Roper et al. 2018; Szuszkiewicz et al. 2022).

During the embryonic pre‐adhesion phase in cows, the embryo secretes a range of biochemical signals, including growth factors, lipids, prostaglandins, and other autocrine embryotropins, which stimulate and prepare the endometrium for adhesion (Wydooghe et al. 2017). We conducted a GSEA analysis in IVF‐ET18 x FTAI18, the cow's embryonic pre‐adhesion period. Members of the *Tgfβ* family (transforming growth factor β) are located at the maternal‐fetal interface and control the expression of integrins and extracellular matrix proteins that promote embryonic attachment and adhesion in women, mouse and cattle (Blitek et al. 2013; Jones et al. 2006; Sehring et al. 2022). In addition, *Tgfβ*1 has been widely implicated in the regulation of maternal immune tolerance to the conceptus, contributing to the establishment of an immunologically permissive uterine environment during early pregnancy (Yang et al. 2021). Extracellular matrix components must be cleaved by proteases to promote adhesion of the embryo to the endometrium (Sehring et al. 2022). *Tgfβ3* is found in bovine caruncular tissue and in the embryo on Days 15–17 of gestation (Adhikari et al. 2022; Mamo et al. 2012). A reduction in *Tgfβ3* expression was observed at Day 18 in pregnant cows compared to nonpregnant cows in the same luteal stage (Musavi et al. 2018). Similarly, in sheep, a reduction in *Tgfβ3* expression was also found at Day 16 of pregnancy, in comparison to nonpregnant ewes at the same stage, suggesting that the presence of the embryo suppresses *Tgfβ3* expression (Doré et al. 1996). In human placental villi explants, increased *Tgfβ3* protein in vitro inhibits trophoblast cell invasion and decreases the secretion of *Mmp9* and *uPA*, proteases that degrade extracellular matrix (Lash et al. 2005). In our work, *Tgfβ3* expression was up‐regulated in IVF‐ET18 (FDR *q*‐value < 0.001). We propose that lower *Tgfβ3* expression in FTAI18 may assist embryo adhesion, while increased *Tgfβ3* could reduce the levels of active proteases, impairing endometrial remodeling and embryo adhesion in IVF‐ET pregnancies.

Claudins are integral components of tight junctions and can function either as sealing proteins that reduce paracellular permeability or as pore‐forming proteins that increase paracellular permeability (Krause et al. 2008; Overgaard et al. 2011). During the receptive window, cell junctions in uterine cells are reorganized, altering cell polarity and facilitating embryo adhesion (Pawar et al. 2013). Members of the cell adhesion molecule (CAMs) pathway, including claudins (*CLDN3* and CLDN4) and occludins (*OCLN),* were down‐regulated (FDR *q*‐value < 0.001) in IVF‐ET32 x FTAI32. In cattle, tissue immunostaining showed increased *CLDN3* and *CLDN4* in luminal and glandular epithelial cells at Day 15 of the estrous cycle, and *CLDN4* is upregulated in the endometrium at Day 18 in pregnant cows x nonpregnant cows (Bauersachs et al. 2006; Chankeaw et al. 2021). In mice, *CLDN3* KO led to a reduction in implantation sites, litter size, and fetal weight, linked to defective decidualization (Grund et al. 2022). In a mice hyperlipidemia model, uterine samples were collected 5 days after embryo transfer, revealing increased expression of *CLDN3* and decreased expression of *CLDN4*, *OCLN*, and *ZO1* (Zhang et al. 2024). In endometrial samples from women with reproductive failure and hyperlipidemia, *CLDN3* was up‐regulated and *CLDN4* down‐regulated during the mid‐luteal phase. These findings indicate that hyperlipidemia compromises the integrity of tight junctions in the endometrium, affecting epithelial barrier function and impairing embryo adhesion and implantation (Zhang et al. 2024). Our results suggest that reduced *CLDN3*, *CLDN4*, and *OCLN* expression in IVF‐ET32 may be associated with altered epithelial remodeling and uterine receptivity. While increased *CLDN3* and *CLDN4* expression has been linked to receptivity in cattle and mice, reduced *CLDN4*, *OCLN*, and *ZO1* in women may relate to implantation failure. We propose that these alterations could potentially influence the uterine environment in a manner that is not favorable for embryo adhesion in the IVF‐ET model, although functional validation would be required to confirm this effect.

Implantation failure can be associated with several factors, including immunological imbalance in the endometrium (Yockey and Iwasaki 2018). Within the cytokine‐cytokine receptor interaction pathways, inflammatory cytokines (*IL1α*, *IL1β*, *IL4, IL9*, *IL13* and *LIF*) were up‐regulated (FDR *q*‐value < 0.001) and anti‐inflammatory cytokines (*IL15*, *IL18* and *IL6*) were down‐regulated (FDR *q*‐value < 0.001) in IVF‐ET32 x FTAI32. *IL4*, *IL6*, and *LIF* are known to play roles in embryo invasion and adhesion in humans (Schäfer‐Somi 2003). In cattle, treatment of bovine endothelial cells with *IL‐6* increases endothelial permeability through the reorganization of actin filaments and alteration in cell shape which, in turn, can impact embryo adhesion and placentation processes (Maruo et al. 2015). In pigs, during the early postimplantation period, increased *IL‐6* expression, along with other mRNAs, appears to be associated with trophoblast and endometrial proliferation and the establishment of maternal‐fetal communication (Modrić et al. 2000). In our study, *IL‐6* was down‐regulated in IVF‐ET32 but up‐regulated in AI32, highlighting its potential role in pregnancy success. Supporting this, previous studies have shown that *IL‐6* supplementation in IVF‐ET embryos increases total cell number, inner cell mass (ICM), placental fluid volume (Days 28–35), and pregnancy rates, reaching levels comparable to those achieved with FTAI in pregnancies terminated at Day 70 (Seekford et al. 2021). Taking together, *IL‐6* plays an essential role in implantation by promoting uterine receptivity, trophoblast proliferation, and modulation of immune response, thus, its reduction observed in IVF‐ET32 may be associated with a less favorable uterine environment for embryo adhesion and pregnancy progression. However, successful implantation requires a finely tuned balance between pro‐inflammatory and anti‐inflammatory signals, particularly during the implantation window, where classically pro‐inflammatory cytokines such as *IL‐6* also contribute to the establishment of uterine receptivity. On the other hand, excessive or dysregulated production of pro‐inflammatory cytokines, such as *IL1β*, can be harmful to the fetus during pregnancy. Löb et al. (2021) evaluated the expression of *IL1β* in the placental tissue of women with spontaneous and recurrent miscarriages and healthy pregnancies at 49–98 days of gestation, and indicated that an increase in *IL1β* is related to a nonreceptive endometrium ending in miscarriages, when compared to healthy pregnancies (Löb et al. 2021). In fact, in IVF‐ET32 x FTAI32, increased *IL1β* and reduced *IL6* expression are observed, which could indicate a more pro‐inflammatory state in IVF‐ET. Based on previous studies, the cargo carried by sEVs can in fact alter inflammatory response, affecting their target‐cells (monocytes and macrophages) (Hosseinkhani et al. 2018; Takenaka et al. 2021). Thus, differences in transcripts present in the sEVs from AI and IVF‐ET may be directly related to maternal‐fetal tolerance.

In regard to lncRNAs and their correlated predicted target mRNAs, we highlight the lncRNA ENSBTAG00000048778 (|Log2(FC)| ≥ + 3.62) and its target *TLR9* (|Log2(FC)| ≥ + 3.41), both up‐regulated in IVF‐ET32 x FTAI32. *TLR9* is involved in the innate immune response and, when activated, triggers the production of pro‐inflammatory cytokines that recruit neutrophils. These neutrophils also release inflammatory mediators, such as *IL‐8* and *TNF‐α*, which contribute to endometrial remodeling and support embryo implantation (Olmos‐ortiz et al. 2019; Williamson et al. 2019). Notwithstanding, a correct balance of inflammatory responses is paramount during early pregnancy, and one such regulatory factor is the SERPIN family, which plays a role in controlling excessive inflammatory processes (Yaron et al. 2021). Notably, several SERPIN family members were enriched within the complement and coagulation cascades pathway in IVF‐ET32 x FTAI32. We observed increased expression of *SERPIND1*, *SERPINC1*, and *SERPINF2*, while *SERPING1*, *SERPINE1*, *SERPINA5*, and *SERPINA1* were significantly down‐regulated (FDR *q*‐value < 0.001). In bovine, tissue immunostaining revealed increased expression of *SERPING1* and *SERPINE1* in stromal epithelial cells on Day 15 of the estrous cycle, suggesting their involvement in endometrial preparation for implantation (Chankeaw et al. 2021). Similarly, in humans, endometrial tissue collected during the implantation window from fertile women showed up‐regulation of *SERPIND1*, *SERPINF2*, *SERPINE1*, *SERPING1*, and *SERPINA1*, supporting their conserved role in modulating endometrial receptivity (Chi et al. 2020). Similar to our findings, a study comparing women who underwent IVF‐ET with those with natural pregnancies also reported increased expression of the *SERPINC1* and decreased expression of the *SERPINE1* in placental tissue collected at 45–50 days of gestation (Zhao, Sun, et al. 2019). Lower expression of *SERPINE1*, *SERPINA5, SERPINA1,* and *SERPING1* and up‐regulation of ENSBTAG00000048778 lncRNA and *TLR9* in IVF‐ET32 x FTAI32 suggest a potential dysregulation of the complement and coagulation signaling pathways and may negatively impact maternal‐fetal communication and the establishment of an adequate uterine environment following IVF‐ET. However, these lncRNA–mRNA regulatory relationships should be interpreted with caution. Although the stringent correlation threshold (|*r*| ≥ 0.99) reduced the likelihood of false‐positive associations, it may also have excluded biologically relevant interactions. Therefore, the identified regulatory relationships should be considered putative and warrant validation in larger datasets and functional studies.

When analyzing the DE miRNAs shared between the gestational time contrasts (18 × 32 days) within the same technique, less than 10% were found to be common. This indicates that miRNA‐mediated posttranscriptional regulation during pregnancy progression occurs differently between IVF‐ET and FTAI. These distinct miRNA profiles are reflected in different biological processes, as evidenced by the pathways of their target genes, which may indicate variations in maternal–fetal communication between the two techniques.

Pathway enrichment analysis using down‐regulated target mRNAs of up‐regulated DE miRNAs in IVF‐ET32 x IVF‐ET18 revealed a significant enrichment of down‐regulated genes in the endocytosis pathway, including *VPS35*, a target of bta‐miR‐2338 (FC = + 1.5 FDR = 0.03), and *VPS36*, targeted by bta‐miR‐2422 (FC = + 1.5 FDR = 0.02) and bta‐miR‐214 (FC = + 1.5 FDR = 0.04). In addition, VPS36 was found to participate in lncRNA–miRNA–mRNA interactions involving three lncRNAs: ENSBTAG0000008296 (|Log2(FC)| ≥ −1.56), ENSBTAG0000052151 (|Log2(FC)| ≥ −2.22), and ENSBTAG0000051191 (|Log2(FC)| ≥ −1.61). These genes are involved in cargo sorting, endosomal trafficking, and small sEVs biogenesis, processes essential for intercellular communication. In bovine embryos, produced by IVF‐ET and parthenogenetic activation, sEVs released between Days 9 and 11 of culturing were evaluated. IVF‐ET‐derived embryos that arrested their development produced a significantly higher amount of sEVs, suggesting that sEV release may be associated with embryonic state and developmental competence, and may also reflect altered or stress‐related cellular activity (Mellisho et al. 2017). In model species, loss of *VPS35* function in neural sEVs led to protein accumulation in recycling endosomes in *Drosophila*, promoting increased incorporation of proteins into sEVs as a compensatory mechanism (Walsh et al. 2021). Additionally, *VPS36* knockout in yeast sEVs resulted in decreased protein content and increased vesicle size (Zhao, Bleackley, et al. 2019). Although these studies were performed in phylogenetically distant model species, they provide insights on how sEV‐mediated communication in IVF‐ET embryos may be dysregulated, as these results are consistent with our findings of reduced sEV size and increased sEV concentration in IVF‐ET32.

Although the present study provides novel insights into maternal‐fetal communication mediated by sEVs in Nelore x Angus crosses, some limitations should be considered. RNA‐seq reads were aligned to the ARS‐UCD1.2 reference genome, derived from a *Bos taurus* animal. Although genomic divergence between *Bos indicus* and *B. taurus* may introduce mapping bias in highly variable regions, the use of *B. taurus* reference genomes for *B. indicus* genomic and transcriptomic analyses is common practice and generally results in high mapping rates (Masharing et al. 2023; Thambiraja et al. 2026). Therefore, while reference‐related bias cannot be completely excluded, its impact on the overall interpretation of the results is likely limited. Future studies using *B. indicus*‐specific reference genomes or pangenome approaches may further reduce potential reference bias.

A relevant increase in the number of DE mRNAs on Days 32 x 18 in IVF‐ET may reflect altered molecular regulation, possibly influenced by transcriptional compensation due to an initial developmental environment that only partially mimics in vivo conditions. The identification of biomarkers related to successful gestation could enable more precise embryo selection prior to embryo transfer, and also offer new approaches, such as the use of sEVs to deliver these biomarkers (Ilahibaks et al. 2023), leading to an improvement in embryo quality and, consequently, increasing IVF‐ET success rates.

## 5 Conclusion

The success of sEVs‐mediated maternal‐fetal communication depends on the cargo they transport. Our study revealed significant differences in the sEVs content of bovine pregnancies produced by IVF‐ET and FTAI. Consistent with the literature, IVF‐ET pregnancies showed a high number of DE transcripts, suggesting biotechnology‐driven alterations. We identified genes encoding cytokines that may impair uterine receptivity and compromise maternal‐fetal tolerance, as well as genes involved in pre and postadhesion. Additionally, genes associated with endocytosis suggest an increase release of sEVs, aligning with our findings of increase sEV concentrations at 32 days of IVF‐ET gestation compared to 18 days.

## Author Contributions

**Amanda de Oliveira Furlan:** investigation, methodology, formal analysis, writing – review and editing, writing – original draft. **Alessandra Bridi:** investigation. **Jéssica Nora Drum:** investigation. **Liza Margareth Medeiros de Carvalho Sousa:** investigation. **Mariana Cordeiro Almeida:** methodology, formal analysis. **Juliana de Souza Felix:** methodology, formal analysis. **Luana Araujo Zutin:** methodology, formal analysis. **Isabelle Alexandrino dos Santos:** methodology, formal analysis. **Flávia Regina Florencio de Athayde:** methodology, formal analysis. **Natália Francisco Scaramele:** investigation. **Camila Azzolin de Souza:** methodology, formal analysis. **Paula de Carvalho Papa:** funding acquisition. **Juliano Coelho da Silveira:** funding acquisition, investigation. **Roberto Sartori Filho:** funding acquisition. **Danila Barreiro Campos:** conceptualization, funding acquisition, writing – review and editing. **Flavia Lombardi Lopes:** conceptualization, funding acquisition, writing – review and editing.

## Conflicts of Interest

The authors declare no conflicts of interest.

## Supporting information

Supporting File 1

Supporting File 2

Supporting File 3

Supporting File 4

Supporting File 5

Supporting File 6

Supporting File 7

Supporting File 8

Supporting File 9

Supporting File 10

Supporting File 11

Supporting File 12

Supporting File 13

## Data Availability

The datasets generated for this study will be made available in the Gene Expression Omnibus (https://www.ncbi.nlm.nih.gov/geo) under accession numbers GSE284769 (RNA‐Seq data) and GSE284542 (microarray data) once the manuscript is accepted for publication.
