# Supplementary material for: Coding and Noncoding Uterine Small Extracellular Vesicle Content Differs in the Early Stages of Pregnancies Produced by Artificial Insemination and In Vitro Fertilization in Cattle
Source: Mol Reprod Dev. 2026 Jul 6;93(7):e70132. doi: 10.1002/mrd.70132 (PMC13334345; doi:10.1002/mrd.70132)
Supplement: Supplementary file 1 — Supporting File 1 [file MRD-93-e70132-s007.docx]

**Supplemental Figure S1**


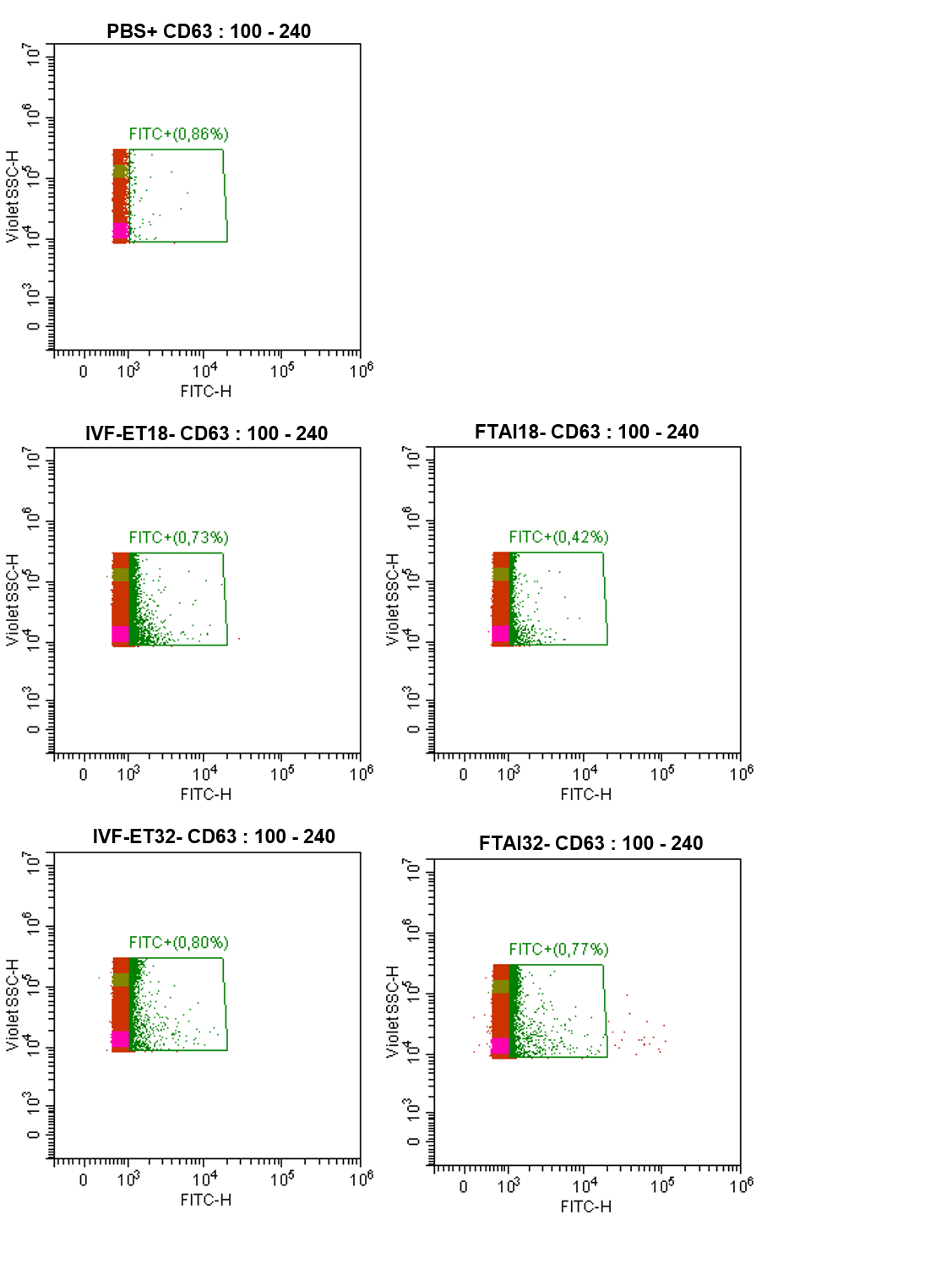


**Figure S1. A)** Characterization of small extracellular vesicles by nano-flow cytometry. Calibration beads (100–300 nm) were used for size gating. CD63 was used as a marker of vesicle membrane proteins, and PBS + ATC served as the control for defining fluorescence-positive events.


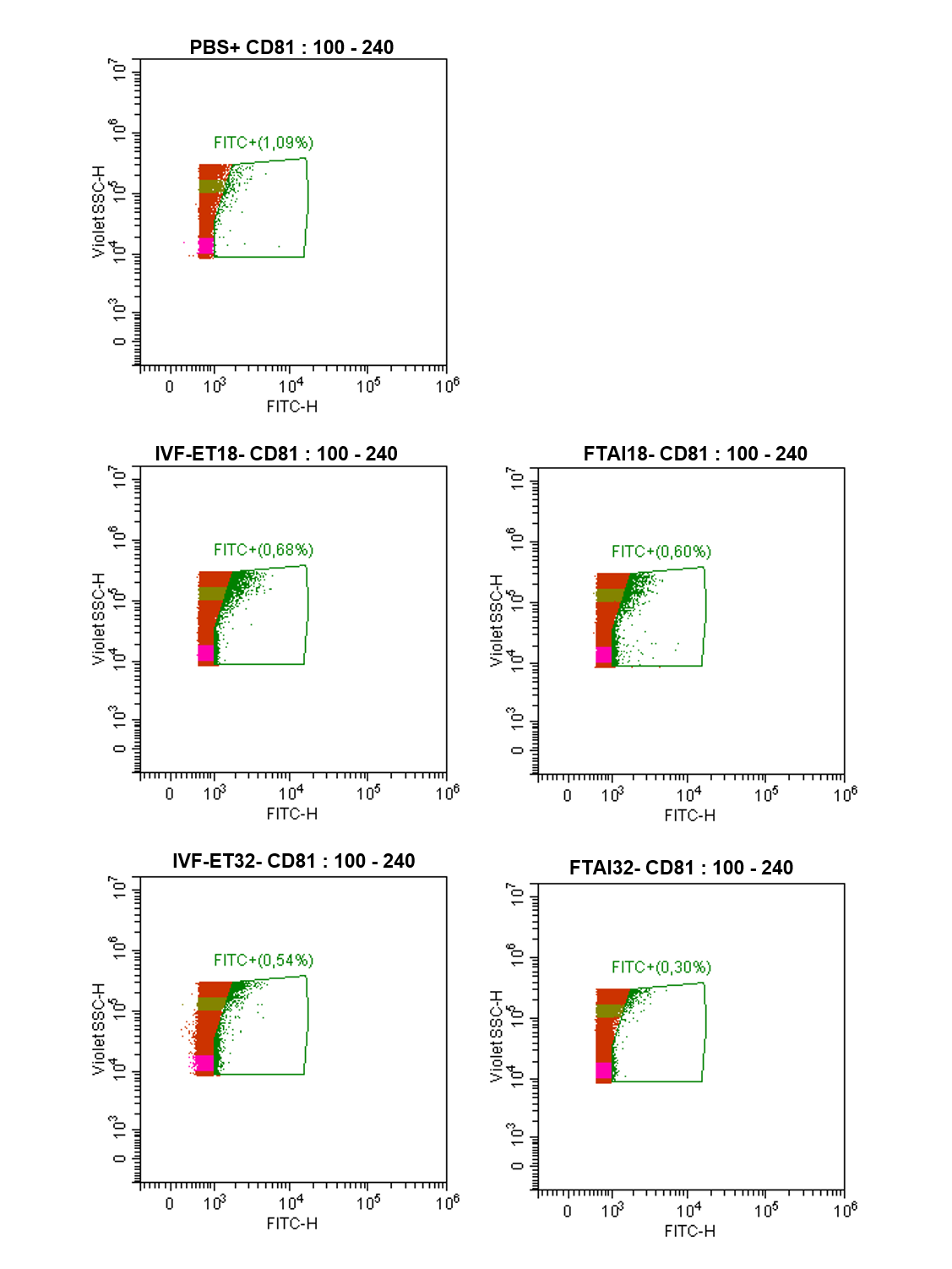


**Figure S1. B)** Characterization of small extracellular vesicles by nano-flow cytometry. Calibration beads (100–300 nm) were used for size gating. CD81 was used as a marker of vesicle membrane proteins, and PBS + ATC served as the control for defining fluorescence-positive events.


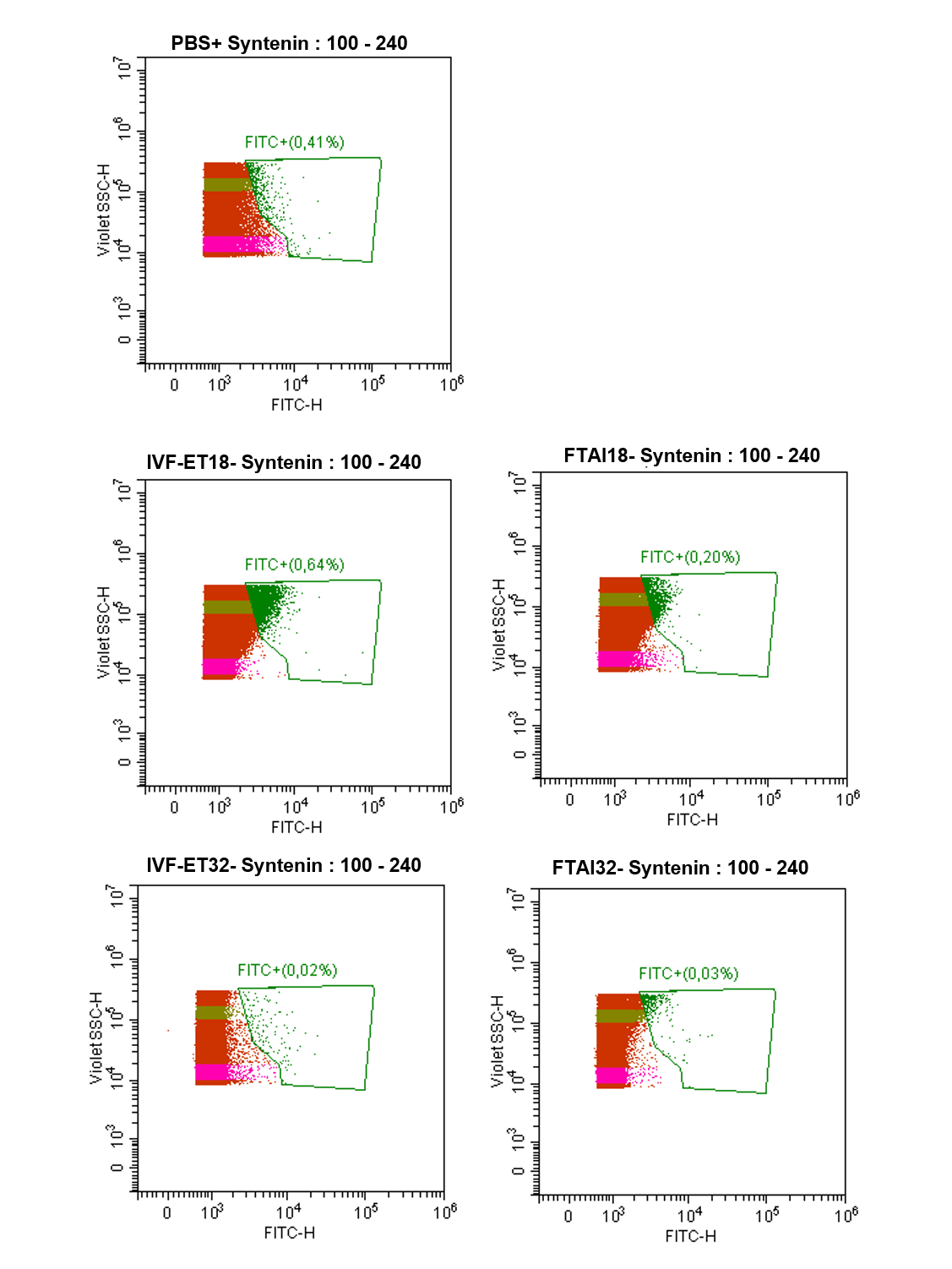


**Figure S1. C)** Characterization of small extracellular vesicles by nano-flow cytometry. Calibration beads (100–300 nm) were used for size gating. Syntenin was used as a marker of vesicle membrane proteins, and PBS + ATC served as the control for defining fluorescence-positive events.


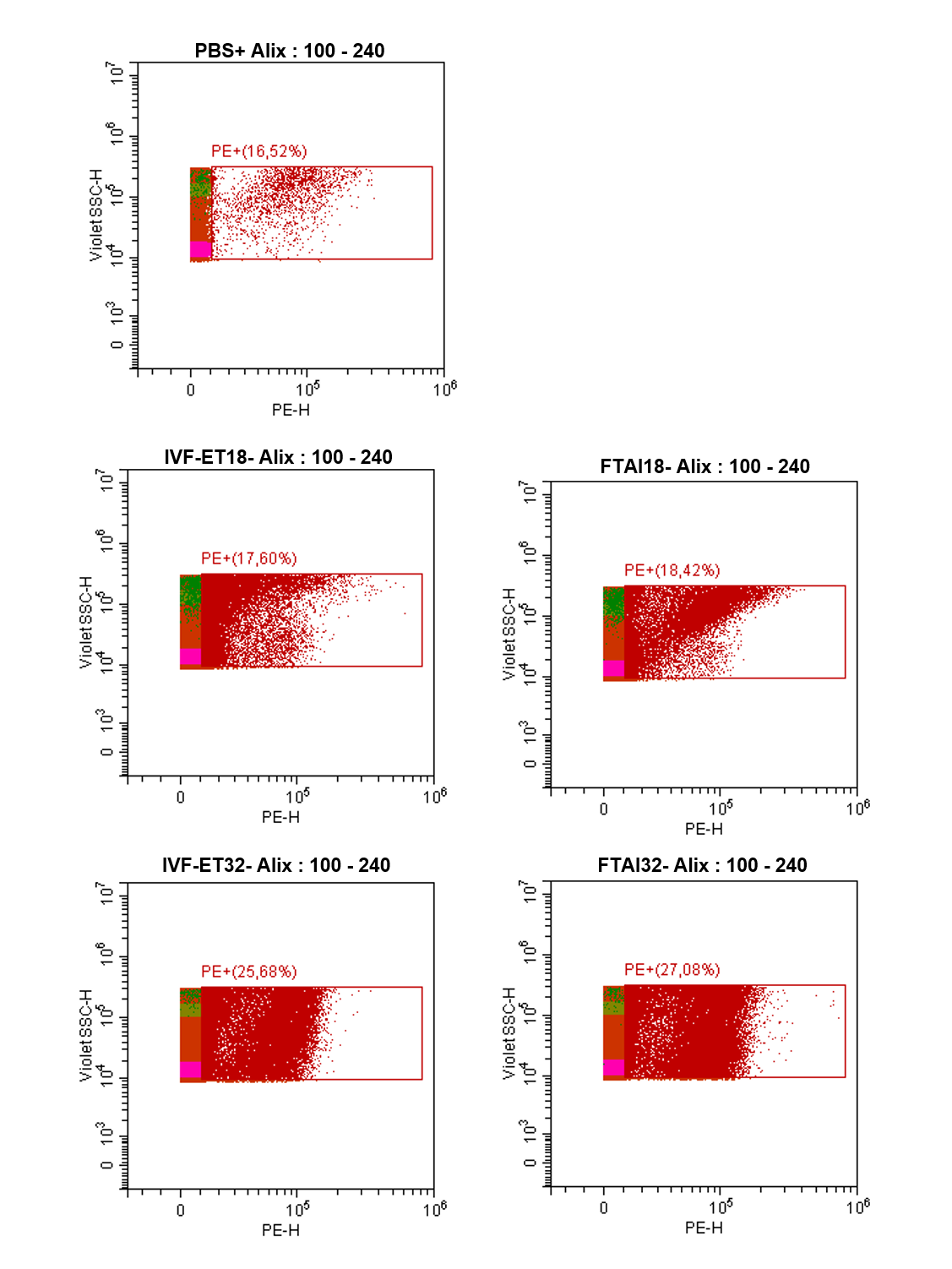


**Figure S1. D)** Characterization of small extracellular vesicles by nano-flow cytometry. Calibration beads (100–300 nm) were used for size gating. Alix was used as a marker of vesicle membrane proteins, and PBS + ATC served as the control for defining fluorescence-positive events.


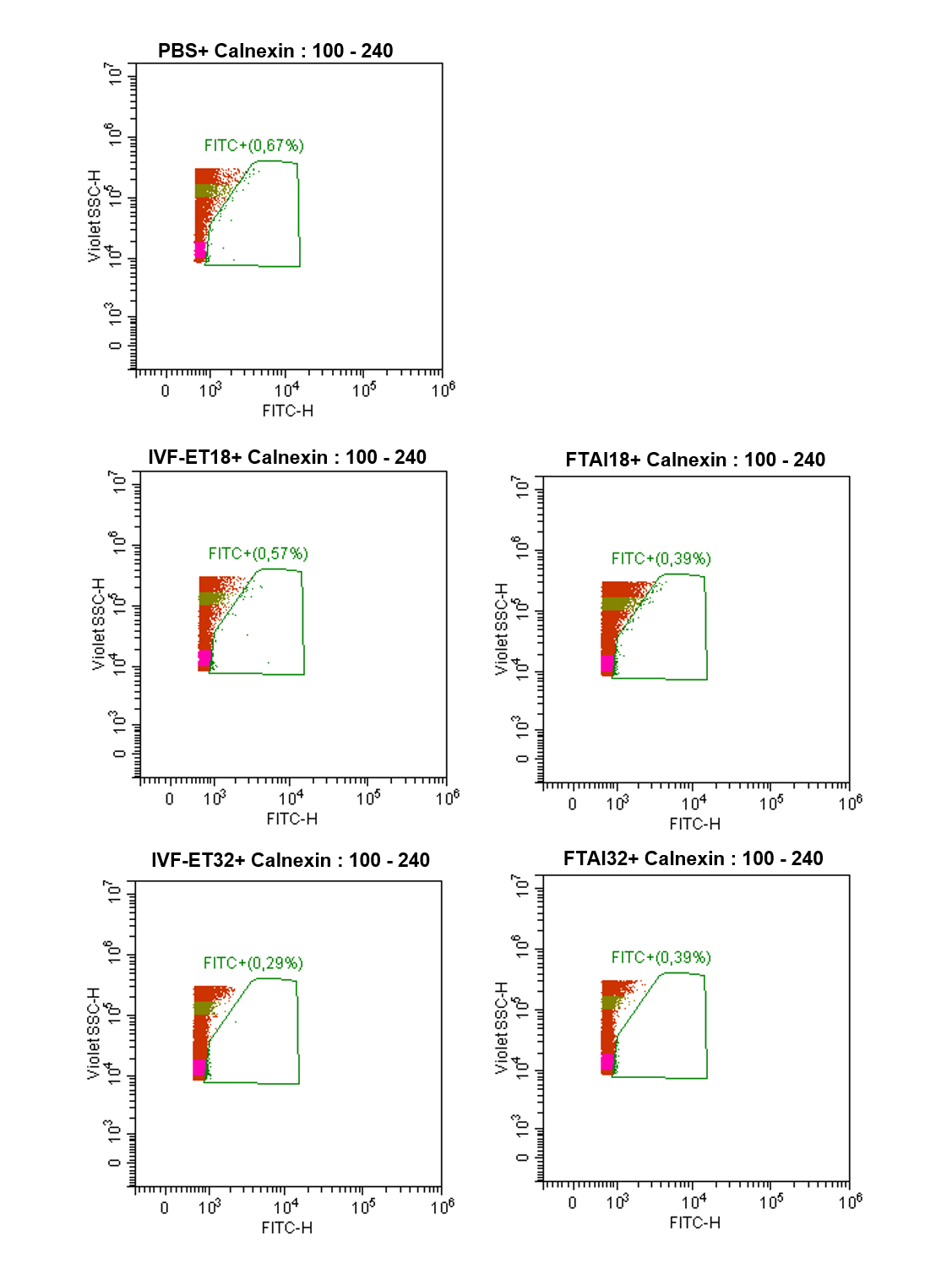


**Figure S1. E)** Characterization of small extracellular vesicles by nano-flow cytometry. Calibration beads (100–300 nm) were used for size gating. Calnexin was used as a marker of vesicle membrane proteins, and PBS + ATC served as the control for defining fluorescence-positive events.
