# Supplementary material for: Coding and Noncoding Uterine Small Extracellular Vesicle Content Differs in the Early Stages of Pregnancies Produced by Artificial Insemination and In Vitro Fertilization in Cattle
Source: Mol Reprod Dev. 2026 Jul 6;93(7):e70132. doi: 10.1002/mrd.70132 (PMC13334345; doi:10.1002/mrd.70132)
Supplement: Supplementary file 2 — Supporting File 2 [file MRD-93-e70132-s008.docx]

**Supplemental Figure S2**


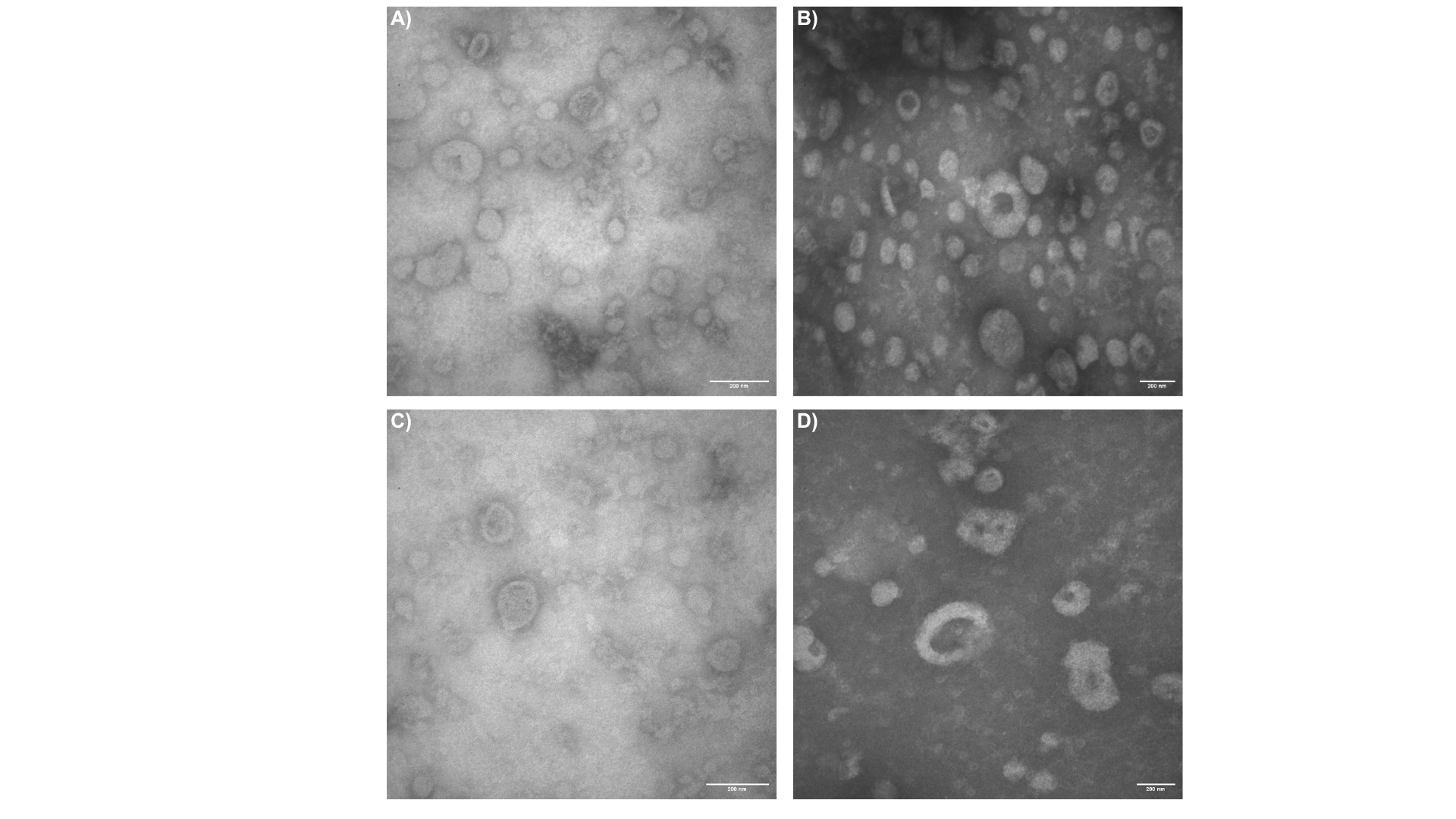


**Figure S2.** Transmission electron microscopy (TEM) was used for morphological analysis (shape and size) of small extracellular vesicles (sEVs) isolated from uterine fluid of FTAI and IVF-ET pregnancies at days 18 and 32 (n = 3 per treatment). **A)** sEVs from FTAI pregnancy at day 18; **B)** sEVs from FTAI pregnancy at day 32; **C)** sEVs from IVF-ET pregnancy at day 18; and **D)** sEVs from IVF-ET pregnancy at day 32.
