# Supplementary material for: Coding and Noncoding Uterine Small Extracellular Vesicle Content Differs in the Early Stages of Pregnancies Produced by Artificial Insemination and In Vitro Fertilization in Cattle
Source: Mol Reprod Dev. 2026 Jul 6;93(7):e70132. doi: 10.1002/mrd.70132 (PMC13334345; doi:10.1002/mrd.70132)
Supplement: Supplementary file 3 — Supporting File 3 [file MRD-93-e70132-s003.docx]

**Supplemental Figure S3**


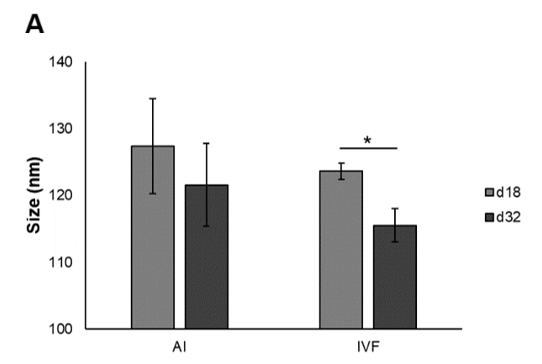


## **Figure S3. A)** Average estimated size of small extracellular vesicles from: (FTAI18) d18 fixed-timed artificial insemination pregnancy; (IVF-ET18) d18 *in vitro* fertilization - embryo transfer pregnancy; (FTAI32) d32 fixed-timed artificial insemination; and (IVF-ET32) d32 *in vitro* fertilization - embryo transfer pregnancy. Data are represented as mean ± standard error.


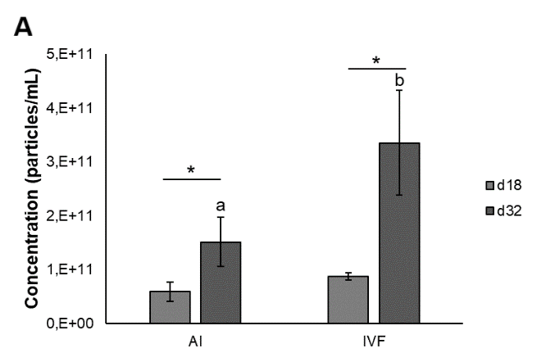


## **Figure S3. B)** Small extracellular vesicles’ concentration in uterine fluid from: (FTAI18) d18 fixed-timed artificial insemination pregnancy; (IVF-ET18) d18 *in vitro* fertilization - embryo transfer pregnancy; (FTAI32) d32 fixed-timed artificial insemination; and (IVF-ET32) *in vitro* fertilization - embryo transfer pregnancy. Data are represented as mean ± standard error.
