# Supplementary material for: Coding and Noncoding Uterine Small Extracellular Vesicle Content Differs in the Early Stages of Pregnancies Produced by Artificial Insemination and In Vitro Fertilization in Cattle
Source: Mol Reprod Dev. 2026 Jul 6;93(7):e70132. doi: 10.1002/mrd.70132 (PMC13334345; doi:10.1002/mrd.70132)
Supplement: Supplementary file 4 — Supporting File 4 [file MRD-93-e70132-s005.docx]

Supplemental Figure S4


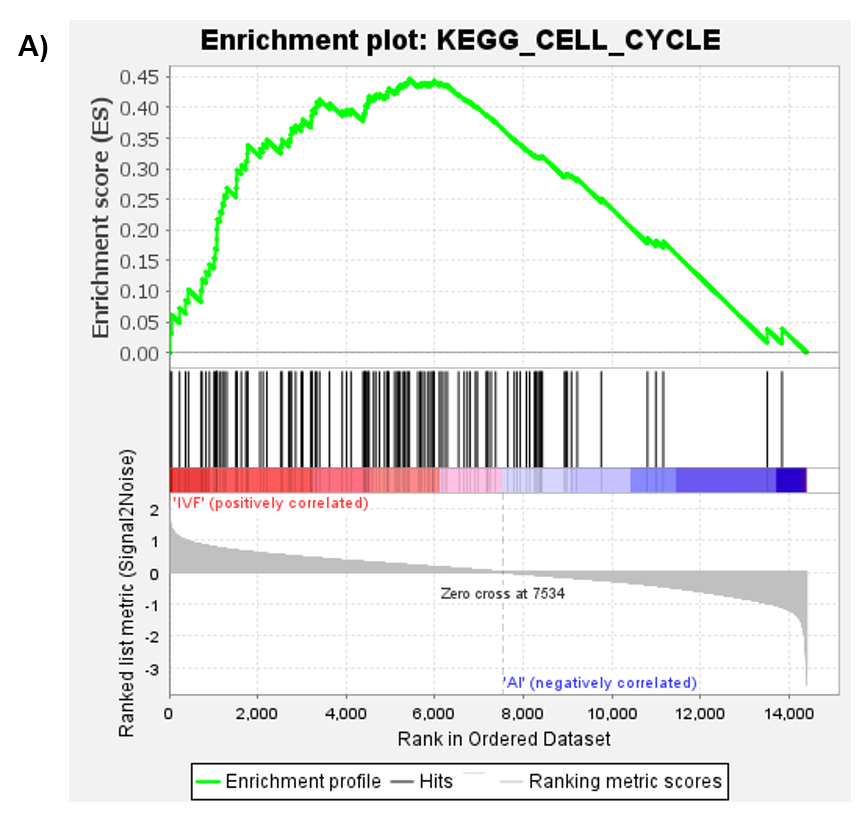


**Figure S4. A)** Gene Set Enrichment Analysis (GSEA) results of IVF-ET18 x FTAI18. KEGG CELL CYCLE enrichment graph. The upper part of the graphs shows the enrichment scores for each gene and the lower part shows the ranked genes. Y-axis: rank metric, X-axis: individual ranks for all genes.


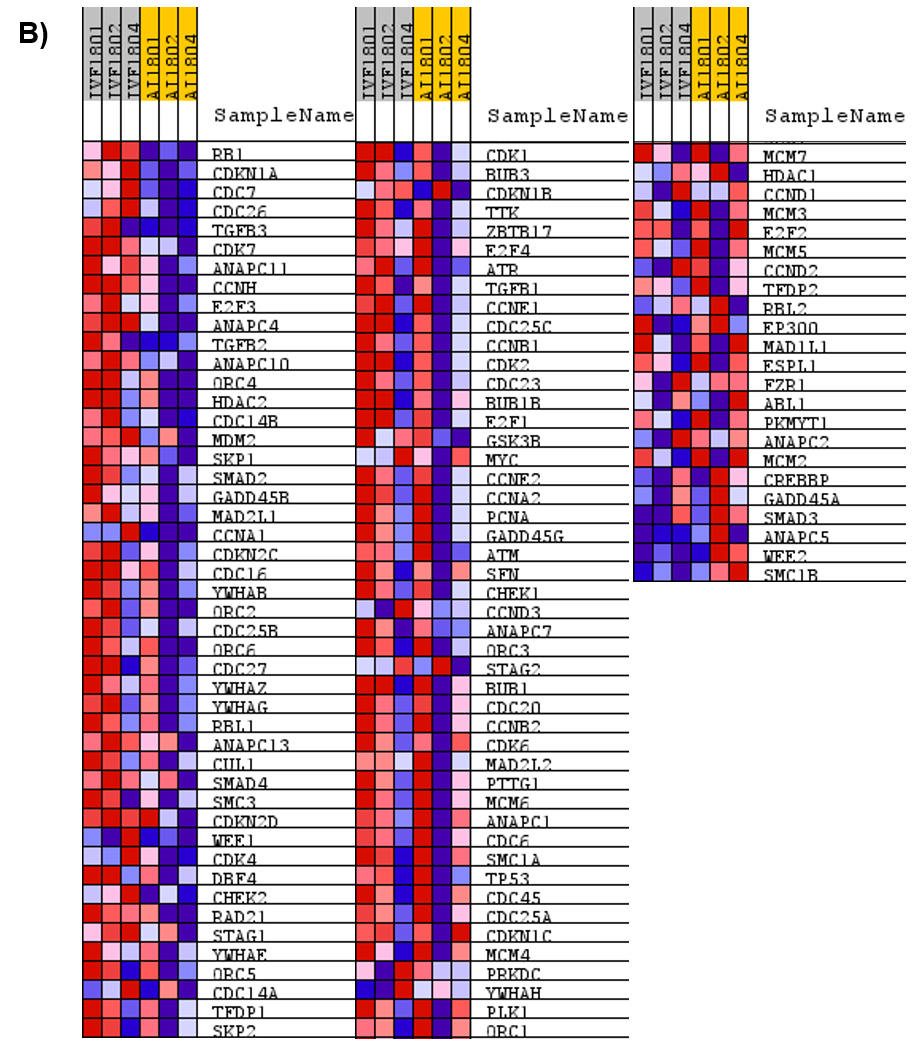


Figure S4. B) Gene Set Enrichment Analysis (GSEA) results of IVF-ET18 x FTAI18. Heatmap showing 117 mRNAs enriched in KEGG CELL CYCLE.
Expression values are represented as colors and range from red (high
expression), pink (moderate), light blue (low expression) to dark blue (lowest
expression). Sample IDs are shown in gray and yellow up on the heatmap.


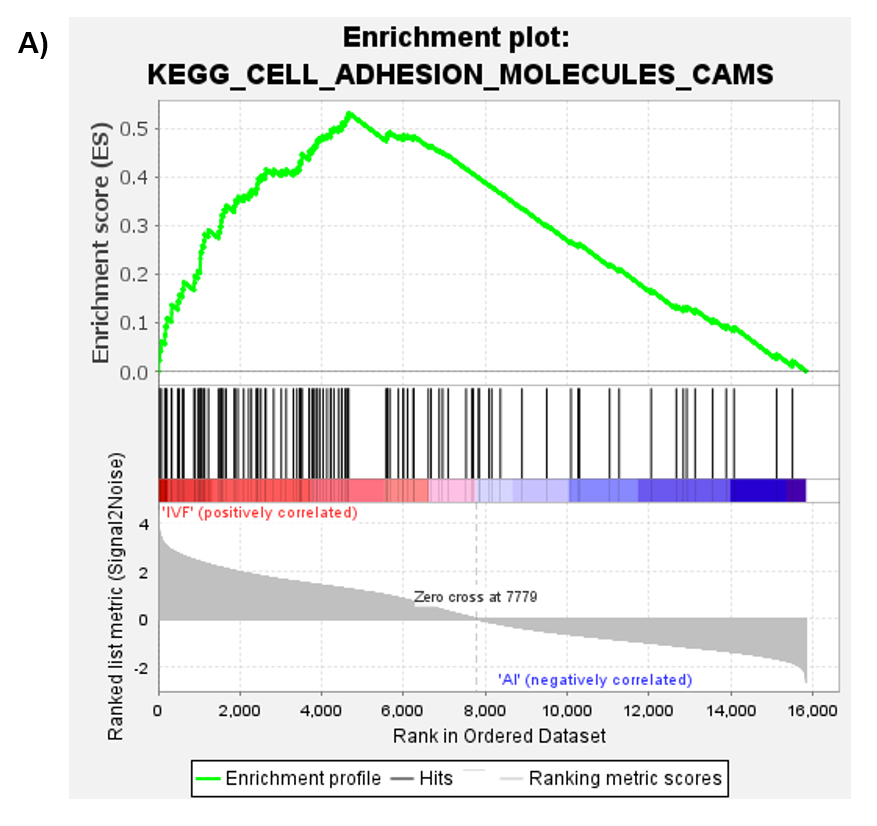


**Figure S4.** **A)** Gene Set Enrichment Analysis (GSEA) results of IVF-ET32 x FTAI32. KEGG CELL ADHESION MOLECULES CAMS enrichment graph. The upper part of the graphs shows the enrichment scores for each gene and the lower part shows the ranked genes. Y-axis: rank metric, X-axis: individual ranks for all genes.


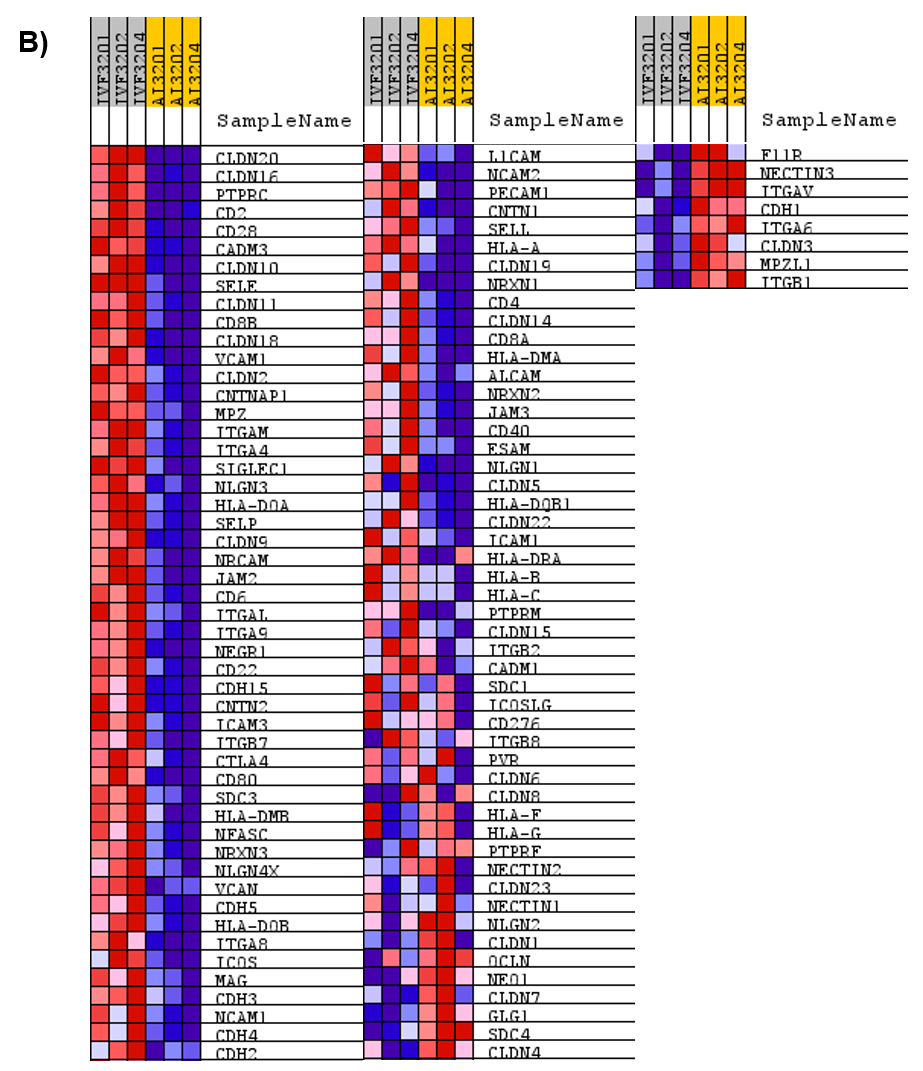


Figure S4. B) Gene Set Enrichment Analysis (GSEA) results of IVF-ET32 x FTAI32. Heatmap showing 108 mRNAs enriched in KEGG CELL ADHESION MOLECULES CAMS. Expression values are represented as colors and range from red (high expression), pink (moderate), light blue (low expression) to dark blue (lowest expression). Sample IDs are shown in gray and yellow up on the heatmap.


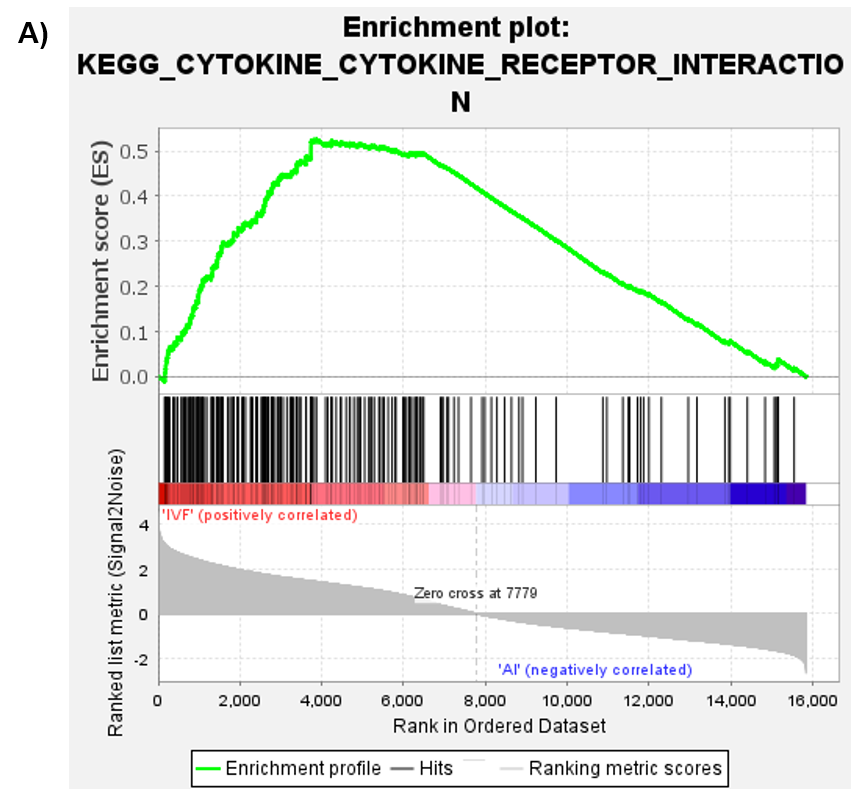


**Figure S4.** **A)** Gene Set Enrichment Analysis (GSEA) results of IVF-ET32 x FTAI32. KEGG CYTOKINE-CYTOKINE RECEPTOR INTERACTION enrichment graph. The upper part of the graphs shows the enrichment scores for each gene and the lower part shows the ranked genes. Y-axis: rank metric, X-axis: individual ranks for all genes.


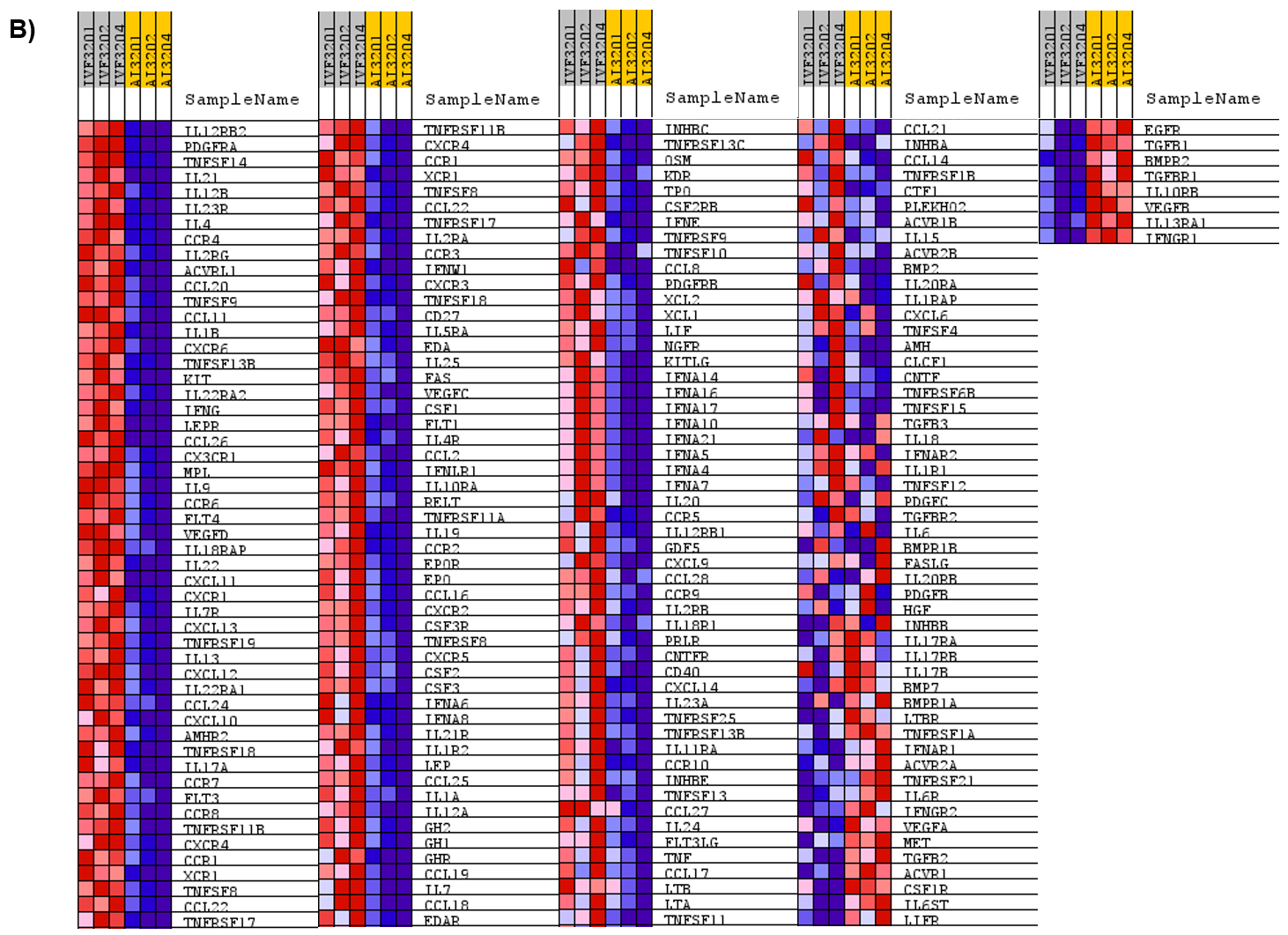


Figure S4. B) Gene Set Enrichment Analysis (GSEA) results of IVF-ET32 x FTAI32. Heatmap showing 209 mRNAs enriched in KEGG CYTOKINE-CYTOKINE RECEPTOR INTERACTION. Expression values are represented as colors and range from red (high expression), pink (moderate), light blue (low expression) to dark blue (lowest expression). Sample IDs are shown in gray and yellow up on the heatmap.


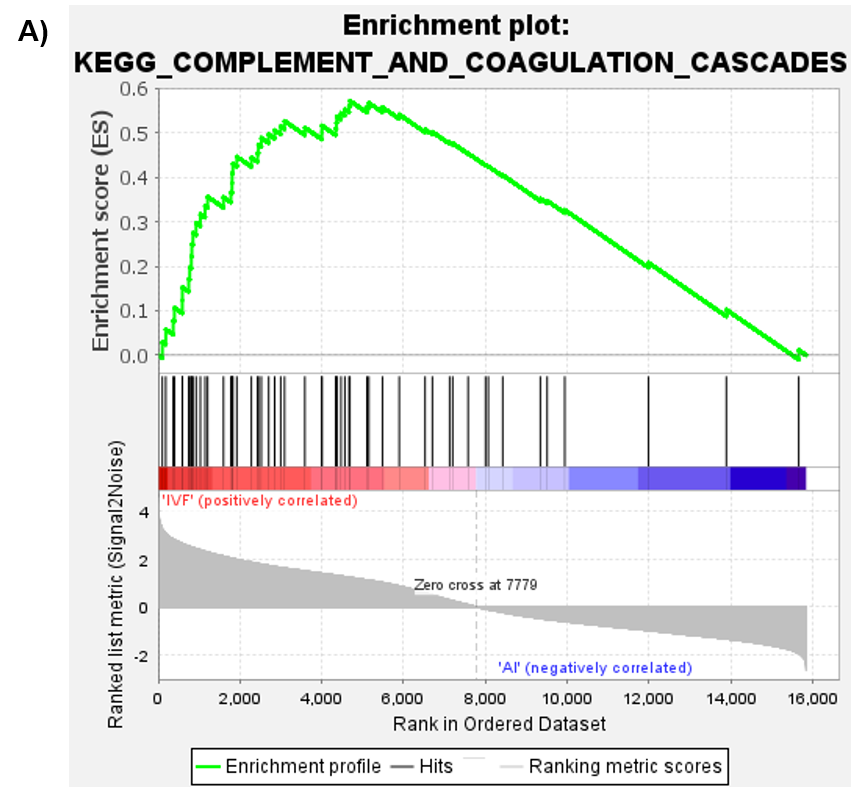


**Figure S4.** **A)** Gene Set Enrichment Analysis (GSEA) results of IVF-ET32 x FTAI32. KEGG COMPLEMENT AND COAGULATION CASCADES enrichment graph. The upper part of the graphs shows the enrichment scores for each gene and the lower part shows the ranked genes. Y-axis: rank metric, X-axis: individual ranks for all genes.


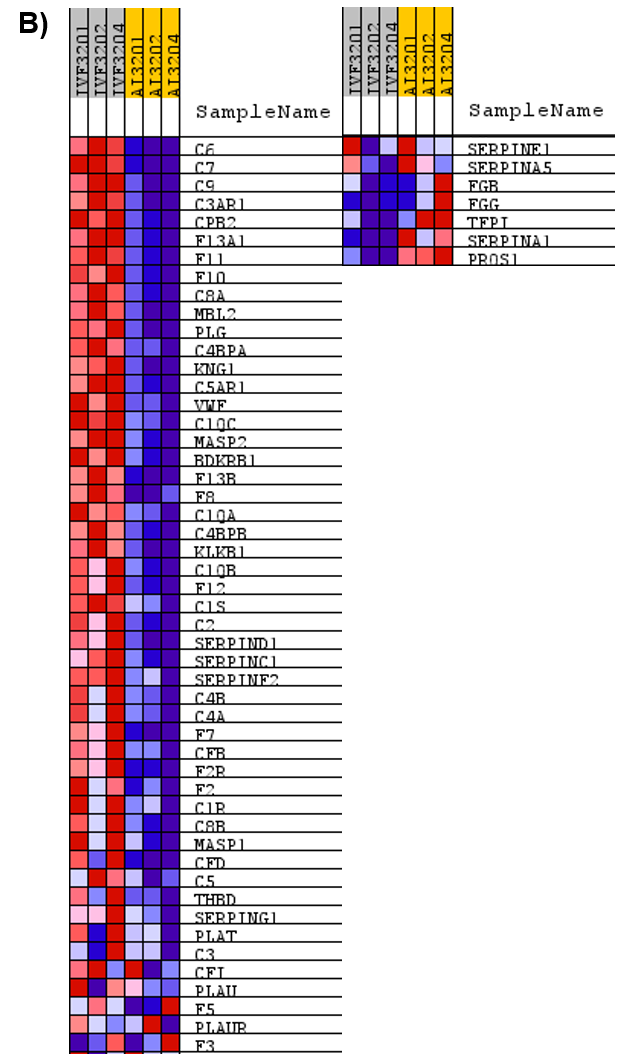


Figure S4. B) Gene Set Enrichment Analysis (GSEA) results of IVF-ET32 x FTAI32. Heatmap showing 57 mRNAs enriched in KEGG COMPLEMENT AND COAGULATION CASCADES. Expression values are represented as colors and range from red (high expression), pink (moderate), light blue (low expression) to dark blue (lowest expression). Sample IDs are shown in gray and yellow up on the heatmap.
